# Mitochondrial uncoupling links lipid catabolism to Akt inhibition and resistance to tumorigenesis

**DOI:** 10.1038/ncomms9137

**Published:** 2015-08-27

**Authors:** Sara M. Nowinski, Ashley Solmonson, Joyce E. Rundhaug, Okkyung Rho, Jiyoon Cho, Cory U. Lago, Christopher L. Riley, Sunhee Lee, Shohei Kohno, Christine K. Dao, Takeshi Nikawa, Shawn B. Bratton, Casey W. Wright, Susan M. Fischer, John DiGiovanni, Edward M. Mills

**Affiliations:** 1 Division of Pharmacology and Toxicology, College of Pharmacy, The University of Texas at Austin, Austin, Texas 78712, USA; 2 Center for Molecular and Cellular Toxicology, College of Pharmacy, The University of Texas at Austin, Austin, Texas 78712, USA; 3 Institute for Cellular and Molecular Biology, The University of Texas at Austin, Austin, Texas 78712, USA; 4 Science Park Research Division, University of Texas MD Anderson Cancer Center, Smithville, Texas 78957, USA; 5 Center for Molecular Medicine, National Heart, Lung, and Blood Institute, National Institutes of Health, Bethesda, Maryland 20892, USA; 6 Department of Nutritional Physiology, Institute of Health Biosciences, Tokushima University Graduate School, Tokushima 770-8503, Japan

## Abstract

To support growth, tumour cells reprogramme their metabolism to simultaneously upregulate macromolecular biosynthesis while maintaining energy production. Uncoupling proteins (UCPs) oppose this phenotype by inducing futile mitochondrial respiration that is uncoupled from ATP synthesis, resulting in nutrient wasting. Here using a UCP3 transgene targeted to the basal epidermis, we show that forced mitochondrial uncoupling inhibits skin carcinogenesis by blocking Akt activation. Similarly, Akt activation is markedly inhibited in UCP3 overexpressing primary human keratinocytes. Mechanistic studies reveal that uncoupling increases fatty acid oxidation and membrane phospholipid catabolism, and impairs recruitment of Akt to the plasma membrane. Overexpression of Akt overcomes metabolic regulation by UCP3, rescuing carcinogenesis. These findings demonstrate that mitochondrial uncoupling is an effective strategy to limit proliferation and tumorigenesis through inhibition of Akt, and illuminate a novel mechanism of crosstalk between mitochondrial metabolism and growth signalling.

## Abstract (web summary)

Aerobic glycolysis and diminished oxidative phosphorylation exhibited by tumour cells enables the production of energy necessary to support malignant proliferation. Here the authors show that UCP3 promotes mitochondrial uncoupling and prevents tumorigenesis through a mitochondrially-driven pathway of AKT inhibition.

## Graphical abstract

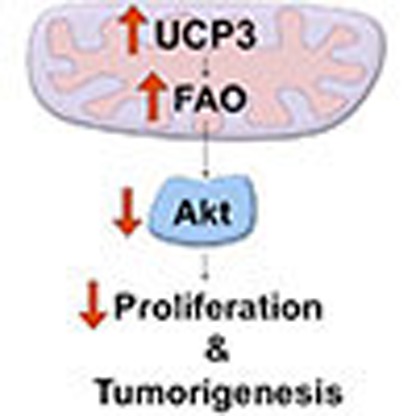

In the last decade, researchers have renewed efforts to understand the nearly century-old observation that tumour cells undergo a metabolic switch, implementing aerobic glycolysis (also known as the Warburg Effect) and suppressing mitochondrial oxidative phosphorylation^1^. We now appreciate that tumour cells’ seemingly paradoxical reliance on the less efficient process of glycolysis for generation of ATP likely confers a growth advantage, by allowing cells to balance the need to produce energy with the need to produce biomass. In addition to upregulating glycolysis, changes in mitochondrial function appear to re-direct nutrient utilization away from substrate oxidation and towards biosynthesis, allowing mitochondria to provide metabolic precursors for the construction of nucleotides, proteins and lipids^2^,^3^. This crucial restructuring of tumour cell metabolism is controlled by classical oncoproteins and tumour suppressors, such as c-myc, hypoxia inducible factors, p53, mammalian target of rapamycin (mTOR), phosphatidylinositol 3-kinase (PI3K) and Akt (protein kinase B)^4^,^5^.

Akt is a serine/threonine kinase that is one of the most commonly upregulated oncoproteins in a variety of cancers. Activated in response to mitogens such as epidermal growth factor (EGF), Akt phosphorylates a myriad of proteins involved in protein translation, metabolism, cell survival/anti-apoptosis and cell cycle progression, including among others glycogen synthase kinase-3α/β (GSK-3α/β), Forkhead box protein O (FOXO) transcription factors, tuberin/tuberous sclerosis 2 (TSC2), p21 (Cip1/Waf1) and p27 (Kip1; refs 6, 7). Simultaneously, Akt controls proliferation in part by driving metabolic reprogramming characterized by increased glucose uptake^8^, glycolytic rate and lactate production^9^, and *de novo* lipid synthesis^10^, along with suppression of macromolecular degradation^11^,^12^. In addition to the regulation of cellular metabolism by growth signalling, metabolic changes are well known to exert reciprocal control of growth signalling pathways (for example, mTOR)^4^. In contrast to its well-established role in metabolic regulation, there is a paucity of information regarding whether and how metabolic state modulates Akt signalling.

One effective strategy to drive nutrient oxidation and limit energy production is to decrease the efficiency of mitochondrial oxidative phosphorylation through proton leak. Mitochondrial uncoupling proteins (UCPs) are evolutionarily conserved, nuclear-encoded members of the mitochondrial solute carrier superfamily that increase proton leak across the inner mitochondrial membrane in a variety of tissues, including brown fat, heart, skeletal muscle and most recently, skin^13^,^14^,^15^. Also referred to as mitochondrial uncoupling, the proton leak generated by UCPs disengages fuel oxidation and electron transport from ATP synthesis, and as a result, uncoupled cells increase substrate oxidation and electron transport in an effort to maintain mitochondrial membrane potential^16^,^17^. Given that mitochondrial uncoupling drives catabolic metabolism, contrary to the anabolic needs of cancer cells, we hypothesized that driving this process would oppose tumorigenesis. As proof of principle, we previously published that overexpression of UCP3 blocked chemically mediated skin carcinogenesis^18^. Here we show that the mechanism underlying uncoupling-induced resistance to tumour formation involves the marked inhibition of tumour promotion through blockade of PI3K/Akt signalling. We demonstrate that enforced mitochondrial uncoupling enhances lipid catabolism and membrane phospholipid breakdown, causing the accumulation of lysophospholipid by-products, and restricting Akt membrane recruitment and activity. Furthermore, overexpression of wild-type Akt in the presence of UCP3 rescues two-stage chemical carcinogenesis. These results establish a mechanism for UCP3-induced chemoresistance, and identify a unique pathway of metabolic Akt regulation that could be exploited by future therapeutic strategies.

## Results

### UCP3 expression blocks tumour promotion

We previously generated *K5-UCP3* mice that overexpress murine UCP3, targeted to the basal epidermis by the bovine keratin 5 promoter (K5; ref. 18). *K5-UCP3* basal keratinocytes exhibit proper localization of UCP3 in the mitochondria, and compared with wild type, *K5-UCP3* epidermis exhibits an approximately twofold increase in uncoupled per total respiration, indicating that the transgenic mitochondria are uncoupled, and the *K5-UCP3* transgene is functional^18^. To further validate this observation, we quantified mitochondrial membrane potential in isolated primary keratinocytes using tetramethylrhodamine methyl ester (TMRM) staining, and flow cytometry. Staining with the non-potentiometric indicator MitoTracker Green (MTG) was used as a control to normalize for increases in mitochondrial mass, as previously described (*ibid*). This analysis revealed decreased mitochondrial membrane potential in *K5-UCP3* primary keratinocytes (Supplementary Fig. 1a). Furthermore, we observed a 20% decrease in ATP levels in *K5-UCP3* isolated primary keratinocytes compared with wild type (Supplementary Fig. 1b). Taken together, these experiments confirmed that *K5-UCP3* mitochondria display hallmarks of increased uncoupling, in agreement with previously published observations^18^.

Using a two-stage chemical carcinogenesis regimen, we previously showed that *K5-UCP3* mice were exceptionally resistant to skin tumour formation^18^. In support of the idea that this resistance resulted from mitochondrial uncoupling, we performed complementary analyses in mice engineered to ectopically express the prototypical UCP1, also targeted to the basal epidermis (*K5-UCP1*). *K5-UCP1* mice were also resistant to tumour formation in response to a two-stage chemical carcinogenesis regimen (Supplementary Fig. 1c-f), strongly suggesting that resistance to tumorigenesis in these transgenic models resulted from mitochondrial uncoupling.

**Figure 1:**
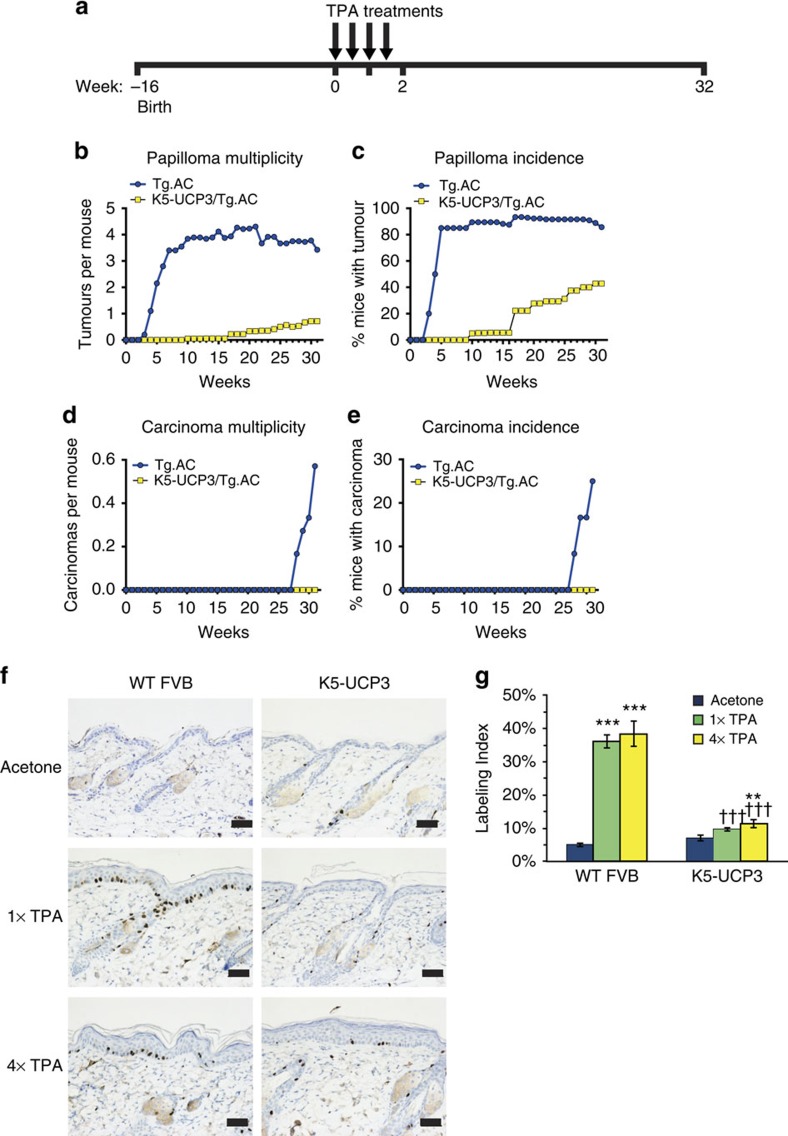
UCP3 overexpression impedes tumour promotion. (**a**) Timeline (in weeks) for *Tg.AC* tumour experiment. Sixteen-week old mice (*Tg.AC*, *n*=20, *K5-UCP3*/*Tg.AC*, *n*=20) were shaved dorsally and treated with bi-weekly applications of TPA (2.5 μg) for 2 weeks for a total of four treatments. Week ‘0’ indicates first TPA application. (**b**) Tumour development in ‘pre-initiated’ *Tg.AC* and bigenic *K5-UCP3*/*Tg.AC* mice indicating total papillomas per mouse, (**c**) % mice bearing papillomas, (**d**) total carcinomas per mouse, and (**e**) % mice bearing carcinomas. (**f**) Immunohistochemistry for BrdU labelled cells in wild-type FVB and *K5-UCP3* epidermis following topical treatment with acetone (vehicle control), single (1 × ) or multiple (4 × ) treatments with 2.5 μg TPA. Scale bars, 50 microns. (**g**) Quantification of percentage of BrdU-positive labelled cells in the basal layer of the interfollicular epidermis. Error bars represent means+/−s.e.m. (*n*=3 animals per group). *Indicates significantly different from acetone control, same genotype (**P<0.01, ***P<0.0001, one way analysis of variance followed by Dunnett’s *post hoc* analysis), † indicates significantly different from wild-type FVB, same treatment (†††*P*<0.0001, Student’s *t*-test).

To test whether UCP3 confers resistance to tumorigenesis mainly through effects on tumour initiation or tumour promotion, and to test the effect of UCP3 overexpression on tumour formation in a genetic model, we crossed *K5-UCP3* animals with ‘pre-initiated’ *Tg.AC* mice, which harbour an oncogenic *v-Ha-Ras* transgene^19^. In response to topical application of the tumour promoter 12-O-tetradecanoylphorbol-13-acetate (TPA) (treatment timeline Fig. 1a), *Tg.AC* mice rapidly formed tumours; however, bi-transgenic *K5-UCP3*/*Tg.AC* mice phenocopied the potent resistance to tumour formation observed in the *K5-UCP3* background (Fig. 1b–e), indicating that UCP3 overexpression likely interferes with tumour promotion.

Consistent with this phenotype, 5-bromo-2-deoxyuridine (BrdU) labelling revealed that UCP3 overexpression dramatically reduced the proliferative response to both single (1 × ) and multiple (4 × ) TPA treatments by ∼80% (Fig. 1f). Whereas both 1 × and 4 × TPA treatment induced a 7.3–7.8-fold increase in labelling index in wild-type *FVB/N* epidermis compared with acetone (vehicle control), labelling index in *K5-UCP3* epidermis only increased by 1.37 and 1.62-fold in response to 1 × and 4 × TPA treatment, respectively (Fig. 1g).

### UCP3 expression inhibits Akt activation

**Figure 2:**
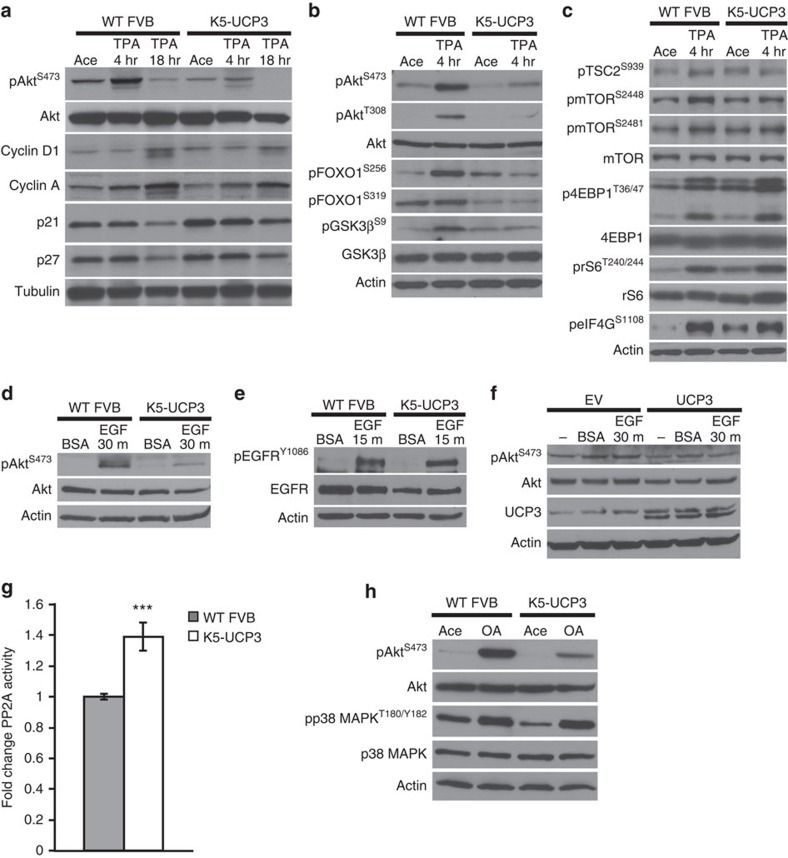
UCP3 overexpression inhibits Akt activation. (**a**) Immunoblot for Akt phosphorylation at Ser 473, along with total Akt, Cyclin D1 and Cyclin A expression, and cell cycle inhibitory proteins p21 (Cip1/Waf1) and p27 (Kip1) expression following treatment with acetone (vehicle control, 4-h post treatment) or 2.5 μg TPA (4 and 18-h post treatment) in wild-type FVB and *K5-UCP3* epidermal lysates. (**b**) Akt phosphorylation at Ser 473 and Thr 308, along with phosphorylation of Akt targets FOXO1 at Ser 256 and Ser 319, and GSK3β at Ser 9 in wild-type FVB and *K5-UCP3* epidermal lysates, following topical treatment with 2.5 μg TPA or acetone. (**c**) Phosphorylation of mTORC1 pathway members TSC2 (Ser 939), mTOR (Ser 2448, Ser 2481), 4EBP1 (Thr 36/47), ribosomal S6 (Thr 240/244) and eIF4G (Ser 1108) in wild-type FVB and *K5-UCP3* epidermal lysates following topical treatment with 2.5-μg TPA or acetone. (**d**) Phosphorylation of Akt Ser 473 and (**e**) phosphorylation of EGFR (Ser 1086) in serum starved wild-type FVB and *K5-UCP3* primary epidermal keratinocytes, after treatment with 40 ng ml^−1^ EGF or 0.001% BSA (vehicle control) at the indicated time points. (**f**) Phosphorylation of Akt Ser 473 in primary neonatal human keratinocytes transiently transfected with UCP3 or empty vector control (EV) and treated with 40 ng ml^−1^ EGF or 0.001% BSA. Immunoblot for UCP3 confirmed successful transfection. (**g**) *In vitro* PP2A catalytic activity, expressed as fold change compared with wild type. Immunoprecipitated PP2Ac was incubated with a target phosphopeptide and free phosphate release was measured using a malachite green assay and absorbance at 620 nm. Error bars represent means+/−s.e.m. (*n*=4 biological replicates). ***Indicates significantly different from wild type (*P*<0.001, Student’s *t*-test). (**h**) Immunoblot for Akt Ser 473 and p38 MAPK T180/Y182 phosphorylation in wild-type FVB and *K5-UCP3* epidermal lysates 1 h following topical treatment with 5-nmol okadaic acid or acetone (vehicle control). β-Actin or α-Tubulin confirmed equal loading (**a-f**,**h**).

Following TPA treatment, activation of numerous cell growth signalling pathways converges at the induction of cyclins and suppression of cell cycle inhibitory proteins to induce cell proliferation^7^,^20^. Consistent with the observed lack of TPA-induced proliferation in *K5-UCP3* epidermis, 18 h after TPA treatment, *K5-UCP3* epidermis showed blunted cyclin D1 and cyclin A induction, and maintenance of p21 and p27 levels (Fig. 2a). To further understand which pathway(s) were involved in this effect, we performed a wide analysis of growth signalling pathways in *K5-UCP3* epidermis via western blotting. Many pathways, including β-catenin, STAT3 and JNK, maintained activation levels similar to that seen in wild-type epidermis (Supplementary Fig. 2a,b). However, activation of Akt was markedly inhibited in *K5-UCP3* epidermis, as indicated by decreased phosphorylation at both S473 (mTORC2 site) and T308 (PDK1 site) compared with wild-type epidermis (Fig. 2a,b). This effect corresponded to decreased phosphorylation of direct Akt targets, including GSK-3β and FOXO1 (Fig. 2b), as well as TSC2 (Fig. 2c).

Given the well-established relationships between metabolic state and mTOR activation, along with the observed lack of Akt activation, we predicted that UCP3 overexpression should correspond to decreased activation of mTOR. Surprisingly, however, mTOR phosphorylation at S2448 was either unchanged or slightly diminished, and mTOR auto-phosphorylation at S2481 was unaffected. Furthermore, phosphorylation of targets downstream of mTORC1, including ribosomal protein S6 (rS6), eukaryotic translation initiation factor 4E-binding protein 1 (4EBP1), and eukaryotic initiation factor 4G (eIF4G) was not markedly decreased, implying that UCP3 induces complex metabolic regulation of mTOR (Fig. 2c). The ability of TPA to activate mTOR despite the absence of Akt activity in *K5-UCP3* epidermis is perhaps somewhat paradoxical, and warrants further investigation into possible alternative mechanisms of mTOR activation in response to TPA treatment. Although these effects on mTOR are interesting, the data point toward an mTOR-independent mechanism of resistance to tumorigenesis in *K5-UCP3* mice because activation of mTOR and its target proteins is similar between wild type and *K5-UCP3* epidermis.

TPA acts in large part as a diacylglycerol mimetic, binding protein kinase C (PKC) isoforms and activating subsequent downstream signalling, which, among numerous other effects, increases EGF ligand expression and ectodomain cleavage, resulting in paracrine/autocrine signalling^21^,^22^. Studies using both chemical inhibitors and dominant negative mutants have shown that EGF receptor (EGFR) activation is necessary for TPA-induced activation of Akt and other pathways^23^,^24^,^25^. To rule out the possibilities that UCP3 overexpression prevented TPA-induced Akt activation via inhibition of PKC and/or impaired EGF ligand shedding, we evaluated Akt activation in serum starved, isolated primary keratinocytes treated with EGF. As expected, *K5-UCP3* cells showed blunted Akt activation after EGF treatment (Fig. 2d). In contrast, EGFR activation was unaffected by UCP3, despite slightly lower total receptor expression (Fig. 2e). Importantly, UCP3 expression also blocked activation of Akt by EGF in primary neonatal human keratinocytes (Fig. 2f). Together, these observations demonstrate that the UCP3-dependent regulation of Akt activity occurs downstream of EGFR activation in a cell autonomous, species-independent manner.

Subsequent to EGFR activation, PI3K converts phosphatidylinositol 4,5-bisphosphate (PIP2) to phosphatidylinositol (3,4,5)-trisphosphate (PIP3), resulting in the recruitment of Akt to the plasma membrane. To define the mechanisms by which UCP3 blunts Akt signalling downstream of EGFR activation, we initially focused on phosphatase and tensin homologue (PTEN), a tumour suppressor lipid phosphatase that converts PIP3 back to PIP2, thereby inhibiting Akt. PTEN activity can be regulated by changes in expression, or in the stabilizing phosphorylation of its C-terminal tail, which increases its activity. Levels of both phosphorylated and total PTEN protein were unchanged in *K5-UCP3* compared with wild-type epidermis, ruling out the likelihood that changes in PTEN function account for UCP3-induced blockade of Akt activation (data not shown).

We then examined another negative regulator of Akt activation, protein phosphatase 2A (PP2A), a serine/threonine phosphatase that dephosphorylates Akt at both S473 and T308 (ref. 26). Although the expression of PP2A A (scaffolding) and C (catalytic) subunits was unchanged from wild type to *K5-UCP3* epidermis (Supplementary Fig. 2c), several additional prominent PP2A targets, including p38-mitogen activated protein kinase (p38 MAPK), showed a pattern of reduced phosphorylation in *K5-UCP3* epidermal lysates (Supplementary Fig. 2d), implying that PP2A was hyper-active in *K5-UCP3* epidermis. Indeed, using an *in vitro* activity assay, *K5-UCP3* samples showed a significant but modest increase in PP2A catalytic activity (39%) compared with wild-type controls (Fig. 2g). Topical treatment with the PP2A inhibitor okadaic acid (OA) was able to completely recover phosphorylation of p38 MAPK, however, OA only partially rescued Akt phosphorylation in *K5-UCP3* epidermis (Fig. 2h). Thus, PP2A hyperactivity likely contributes to, but is not solely responsible for the UCP3-induced blockade of PI3K/Akt signalling. However, the striking effects on other PP2A targets, including p38 MAPK, may inform future studies detailing additional Akt-independent pleiotropic mechanisms of UCP3-induced inhibition of tumorigenesis.

### UCP3 expression alters lipid homoeostasis

Because PP2A hyperactivity could not fully explain the UCP3-dependent inhibition of Akt, we focused on two main functions of UCP3: its ability to decrease reactive oxygen species (ROS) generation^17^, and increase lipid metabolism^27^. Some evidence suggests that ROS regulate both PP2A and Akt^28^,^29^, therefore, we hypothesized that UCP3 overexpression may inhibit Akt through redox regulation. However, we neither detected a significant difference in either cellular ROS or ROS released from isolated mitochondria, nor did we observe a change in the oxidation state of either PP2A or Akt thiols in response to uncoupling (Supplementary Fig. 3).

Because the levels of ROS were similar in wild type and *K5-UCP3* cells and mitochondria, we next used an unbiased metabolomics approach to holistically examine changes in cellular metabolism in *K5-UCP3* epidermis. This wide analysis revealed changes in large classes of nutrient metabolites indicative of increases in several catabolic pathways. We observed decreased levels of six-carbon glycolytic intermediates and increased levels of three-carbon glycolytic intermediates in *K5-UCP3* epidermis, consistent with increased glycolysis (Supplementary Fig. 4a). In contrast, we observed no significant changes in any TCA cycle intermediates, although these steady state data may not be representative of any potential changes in flux through the TCA cycle (Supplementary Fig. 4b). We also observed a trend towards decreased levels of glutamine and glutamate in *K5-UCP3* epidermis, however, this difference was not statistically significant (Supplementary Fig. 4b). These results are intriguing, as tumour cells often also upregulate glycolysis to provide precursors for macromolecular biosynthesis, hence increased glycolysis in a tumour resistant model might seem paradoxical on the surface. However, we also found that UCP3 overexpression resulted in the breakdown of macromolecules necessary for cell proliferation, such as phospholipids.

**Figure 3:**
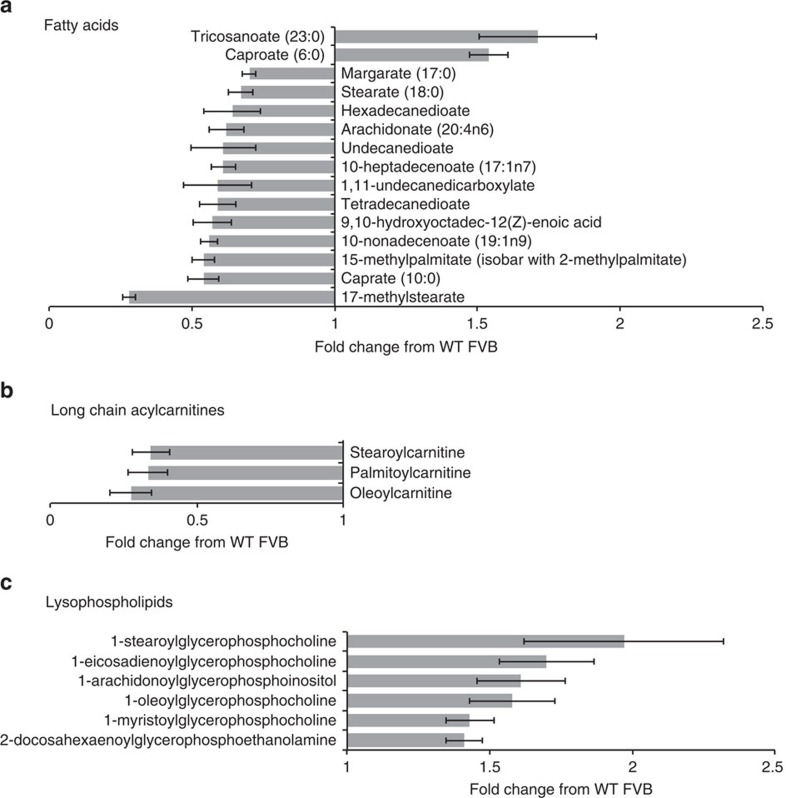
Unbiased metabolomic analysis of *K5-UCP3* epidermis reveals enhanced lipid catabolism. (**a–c**) Analysis of (**a**) fatty acid, (**b**) acylcarnitine and (**c**) lysophospholipid metabolite levels in *K5-UCP3* dorsal epidermis identified by gas chromatography mass spectrometry (GC–MS) or liquid chromatography mass spectrometry (LC-MS), and expressed as fold change compared with wild-type FVB. Metabolites shown significantly differ from wild-type with a *P* value<0.05 (Welch’s *t*-test). Error bars represent means+/−s.e.m. (*n*=6 animals per group). All identified lipids are shown in Supplementary Fig. 5.

**Figure 4:**
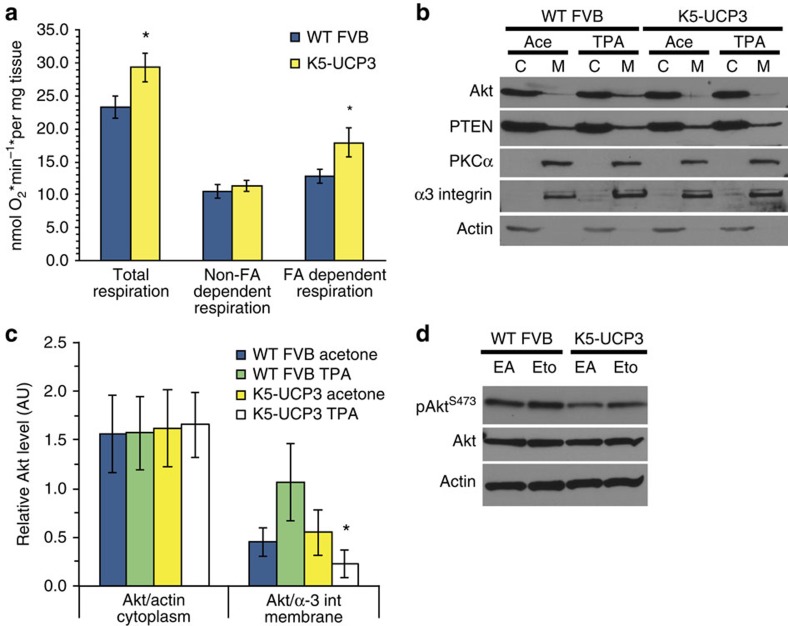
Mitochondrial β-oxidation alters plasma membrane lipids and signaling. (**a**) *Ex vivo* epidermal fatty acid dependent respiration, fatty acid independent respiration and total respiration measured by oxygen consumption in the presence or absence of Etomoxir (Eto) treatment. Fatty acid dependent respiration was defined as the difference between total respiration and Eto inhibited (fatty acid independent) respiration. Error bars represent means+/−s.e.m., (WT FVB *n*=10, *K5-UCP3 n*=8). *Indicates significantly different from wild-type FVB, same treatment (*P*<0.05, Student’s *t*-test). (**b**) Immunoblot for sub-cellular localization of Akt and PTEN in membrane and cytoplasmic fractions from wild-type FVB and *K5-UCP3* epidermis topically treated with 2.5 μg TPA or acetone. α-3 integrin and β-actin were used as controls to verify membrane and cytoplasmic fractions, respectively. (**c**) Relative quantification of Akt in cytoplasmic and membrane fractions from triplicate separate immunoblotting experiments via densitometry. Cytoplasmic and membrane fractions were normalized to β-actin and α3-integrin, respectively. Data represent means+/−s.e.m. (*n*=3 biological replicates). *Indicates significantly different from wild-type FVB, same treatment (*P*<0.05, Student’s *t*-test). (**d**) Immunoblot for Akt Ser 473 phosphorylation in wild-type FVB and *K5-UCP3* epidermal lysates 6 h following topical treatment with 1-mg Eto ethyl ester or ethyl acetate (EA, vehicle control). Immunoblotting for β-Actin was used to confirm equal loading.

Arguably, the largest class effect observed in response to UCP3 expression was increased lipid catabolism in *K5-UCP3* epidermis, characterized by decreased steady-state levels of free fatty acids (Fig. 3a), and long chain acyl-carnitine species (Fig. 3b). Concurrently, *K5-UCP3* epidermis displayed increased levels of lysophospholipids, which are formed by cleavage of a single acyl chain during phospholipid breakdown, suggesting that uncoupled cells scavenge fatty acid tails from membrane lipids as substrates for β-oxidation (Fig. 3c). Consistent with the idea that UCP3 induced global lipid breakdown rather than oxidation of a specific lipid species or activation of a specific phospholipase, neither the decreases in free fatty acids, nor the increases in lysophospholipid levels followed any discernible pattern in terms of chain length, saturation or head group composition (Fig. 3, Supplementary Fig. 5). Although we observed similar levels of total cellular lipid levels in isolated keratinocytes via nile red staining (Supplementary Fig. 6), given the strength of our metabolomics data, we decided to further explore the effect of UCP3 overexpression on lipid metabolism in *K5-UCP3* epidermis. *K5-UCP3* primary keratinocytes showed no change in fatty acid uptake (data not shown) relative to wild-type keratinocytes, but *K5-UCP3* epidermis had significantly increased fatty acid oxidation compared with wild type (Fig. 4a), supporting the notion that *K5-UCP3* keratinocytes scavenge fats from intracellular lipid stores. Similarly, gene expression profiling demonstrated the significant up-regulation of numerous fatty acid metabolism genes in *K5-UCP3* skin (Supplementary Table 1).

Studies have indicated that changes in β-oxidation can reshape plasma membrane lipid composition^30^,^31^, and UCP3 is well known for its ability to increase β-oxidation of lipids^27^. Therefore, we reasoned that UCP3 overexpression might affect Akt activation by promoting the oxidation of fatty acids and modifying membrane composition. To test whether these changes in lipid homoeostasis corresponded to functional differences in membrane recruitment of Akt, we isolated epidermal membranes from acetone and TPA treated mice. As predicted, less Akt was present in the membrane fraction from TPA treated *K5-UCP3* epidermis compared with wild type (Fig. 4b,c). Of note, in the same assay we observed similar levels of Protein Kinase C and PTEN membrane localization in wild type and *K5-UCP3* epidermis, another indication that the activity of these proteins is unchanged (Fig. 4b). Combined, these results suggest the possibility that UCP3-induced lipid catabolism may alter plasma membrane composition and dynamics, thereby impeding Akt membrane recruitment.

To establish the capacity of mitochondrial β-oxidation to affect Akt signalling, we treated mice with the carnitine-palmitoyl transferase inhibitor etomoxir (Eto) at a dose previously shown to inhibit mitochondrial fatty acid uptake by roughly 50% when applied topically^32^. Not only did Eto treatment augment basal Akt activation in *K5-UCP3* epidermis, it also did so in wild-type epidermis, demonstrating that fatty acid oxidation is a novel regulator of Akt signalling (Fig. 4d).

### Akt overexpression rescues tumorigenesis

**Figure 5:**
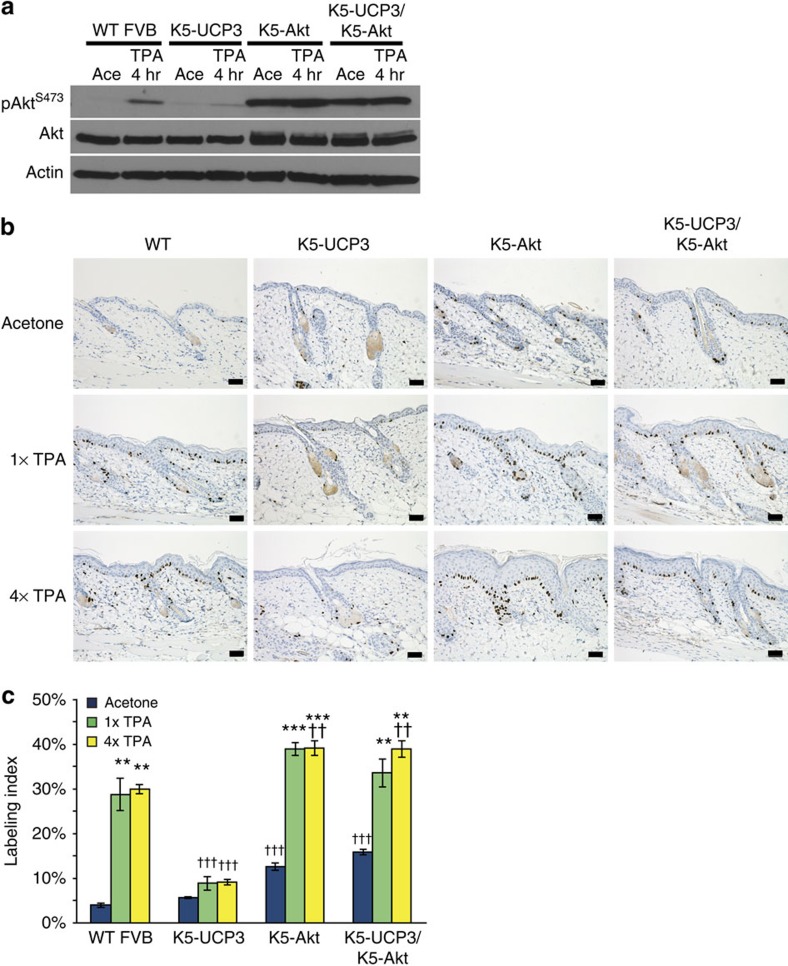
Overexpression of Akt rescues proliferation in *K5-UCP3* epidermis. (**a**) Immunoblot for Akt phosphorylation at Ser 473 in wild-type FVB, *K5-UCP3*, *K5-Akt* and bitransgenic *K5-UCP3*/*K5-Akt* epidermal lysates, 4 h following topical treatment with 2.5 μg TPA or acetone (vehicle control). Immunoblotting for β-Actin was used to confirm equal loading. (**b**) Immunohistochemistry for BrdU labelled cells in wild-type FVB/N, *K5-UCP3*, *K5-Akt*, and bitransgenic *K5-UCP3*/*K5-Akt* epidermis following topical treatment with single (1 × ) or multiple (4 × ) treatments with 2.5 μg TPA or acetone. Scale bars, 50 μ. (**c**) Quantification of BrdU labelled cells in the basal layer of the interfollicular epidermis (IFE). More than 100 cells from five randomly selected skin sections (total >500 cells) were counted from each of *n*=3 mice per genotype in each treatment group. Error bars represent means+/−s.e.m. (*n*=3 animals per group). *Indicates significantly different from acetone control, same genotype (**P<0.01, ***P<0.0001, one way analysis of variance (ANOVA) followed by Dunnett’s *post hoc* analysis), † indicates significantly different from wild-type *FVB/N*, same treatment (††† *P*<0.0001, one way ANOVA followed by Dunnett’s *post hoc* analysis).

To establish the functional importance of UCP3-mediated changes in lipid homoeostasis and Akt activation in UCP3-induced resistance to tumour formation, we inter-bred *K5-UCP3* animals with mice that over-express an epidermally targeted, wild-type Akt transgene (*K5-Akt*)^33^. Akt overexpression had no effect on epidermal respiration (Supplementary Fig. 7); however, as previously published, *K5-Akt* mice exhibited heightened Akt expression and activation compared with wild type controls. Bi-transgenic *K5-UCP3*/*K5-Akt* mice also displayed heightened Akt activation, indicating that Akt overexpression alone was capable of overcoming inhibition by UCP3 (Fig. 5a). In every treatment group, Akt overexpression also increased epidermal proliferation measured by BrdU incorporation, and rescued TPA-induced proliferation in *K5-UCP3*/*K5-Akt* animals (Fig. 5b,c).

**Figure 6:**
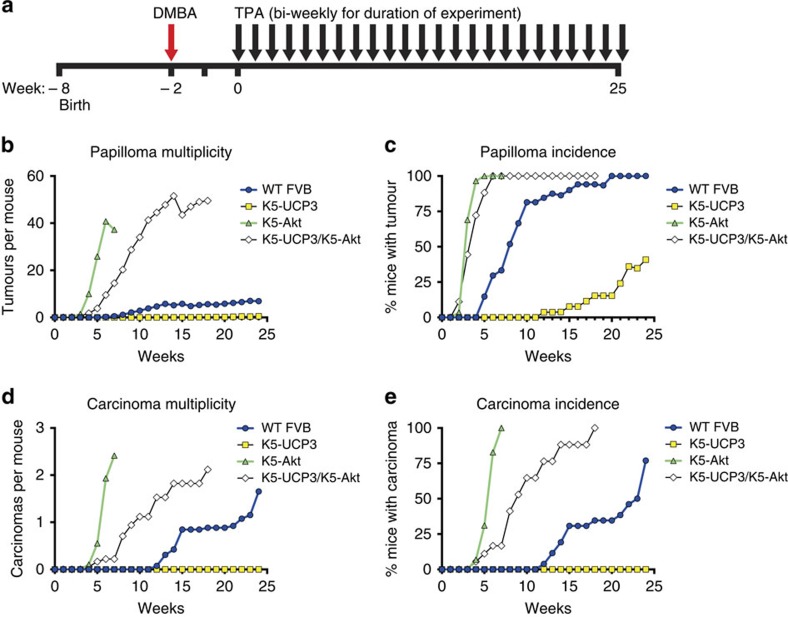
Overexpression of Akt rescues tumorigenesis. (**a**) Timeline for two-stage chemical carcinogenesis tumour experiment. Six-week old mice (WT FVB *n*=28, *K5-UCP3 n*=27, *K5-Akt n*=29, *K5-UCP3*/*K5-Akt n*=18) were shaved dorsally and treated with a single application of DMBA(100 μg) followed 2 weeks later with bi-weekly applications of TPA (2.5 μg) for 26 weeks, as previously described^61^. Week ‘0’ indicates first TPA treatment. (**b**) Tumour development in wild-type FVB, *K5-UCP3*, *K5-Akt*, and bitransgenic *K5-UCP3*/*K5-Akt* mice indicating total papillomas/mouse, (**c**) % mice bearing papillomas, (**d**) total carcinomas/mouse and (**e**) % mice bearing carcinomas.

When subjected to a two-stage chemical carcinogenesis regimen (Fig. 6a), Akt overexpression overcame metabolic regulation and rescued skin tumorigenesis in *K5-UCP3*/*K5-Akt* mice. Bi-transgenic *K5-UCP3*/*K5-Akt* mice and *K5-Akt* single transgenics both formed more papillomas than wild-type mice, and displayed nearly overlapping papilloma incidence curves (Fig. 6b). Papilloma multiplicity revealed a slightly increased latency before papilloma development in *K5-UCP3*/*K5-Akt* animals; however, bi-transgenic mice still formed papillomas more rapidly and abundantly than wild-type littermates (Fig. 6c). *K5-UCP3*/*K5-Akt* mice also formed more carcinomas than wild-type mice, however they still showed a significant reduction in carcinoma formation compared with *K5-Akt* mice (Fig. 6d,e), suggesting that UCP3 may also inhibit tumour progression, even in the context of Akt overexpression. However, this difference may be explained by the fact that the transgene can still be regulated by the same changes that reduce endogenous Akt activation in *K5-UCP3* mice. Notably, no *K5-UCP3* transgenic animal developed a carcinoma in the absence of the *K5-Akt* transgene (Fig. 6d,e), in accordance with our previous findings^18^. Taken together, these findings establish a new mitochondrial pathway of metabolic Akt regulation that results in the profound blockade of tumorigenesis via changes in fatty acid metabolism and membrane homeostasis.

## Discussion

While Otto Warburg originally proposed that mitochondrial defects caused cancer, we now understand that metabolic reprogramming likely confers tumour cells a growth advantage by simultaneously supplying the energy and biomass necessary to rapidly build new daughter cells. Here we show that enforced mitochondrial uncoupling causes resistance to cancer formation, likely as a result of pleiotropic changes in metabolism that converge at least in part on Akt signalling. These results add to a growing number of reports that suggest that manipulation of mitochondrial metabolism and cellular energy balance has profound effects on growth signalling. In contrast to the more well-described pathways through which mitochondria can regulate cellular proliferation through modulation of ATP, ROS and amino-acid metabolism, the lipid-dependent regulation of cell growth signalling is relatively less well understood.

Several indirect lines of evidence support our observation that changes in cellular lipid homoeostasis control proliferation. For example, in *de novo* lipogenesis, citrate is exported from mitochondria and converted to cytoplasmic acetyl CoA by ATP citrate lyase, the rate-limiting enzyme in lipid synthesis. Studies have shown that inhibiting mitochondrial citrate export can block the cell cycle^34^, and knockdown or inhibition of ATP citrate lyase results in inhibition of PI3K/Akt signalling and tumorigenesis^35^,^36^. Other reports have suggested that lipids can affect Akt signalling through bioactive lipid signalling mediators, such as sphingosine-1-phosphate, or through changes in membrane composition and structure^37^,^38^. In addition, lysophospholipids have been shown to affect membrane recruitment and cellular signalling in an array of diverse contexts^39^,^40^, however, the exact mechanisms are not well understood. Future mechanistic studies are needed to further detail how UCP3-induced lipid catabolism affects signalling events and plasma membrane recruitment of Akt. Nonetheless, along with the few reports cited above, our observations lay the foundation and support an urgent need for greater exploration of how lipid metabolism may control cell growth in general.

As noted above, overexpression of Akt in *K5-UCP3*/*K5-Akt* mice rescued and augmented Akt phosphorylation beyond wild-type levels. This suggests that the potential for Akt activation is still intact in the presence of UCP3 overexpression. Several possibilities exist to explain this observation. Despite the fact that the *K5-Akt* transgene is a wild-type copy of Akt, we observed strongly increased Akt phosphorylation in both *K5-Akt* and *K5-UCP3*/*K5-Akt* epidermis even in the absence of TPA treatment. Thus, the overexpression of Akt alone appears to overcome stoichiometry at the plasma membrane, resulting in increased Akt activation even in the absence of increased PIP3 signal in both *K5-Akt* and *K5-UCP3*/*K5-Akt* epidermis. Another possibility is that the elevated expression and phosphorylation of Akt may overcome the PP2A hyperactivity we report in Fig. 2g. Finally, while Akt overexpression fully rescued Akt phosphorylation, BrdU incorporation and papilloma formation, we still observed increased latency in papilloma formation, and modest decreases in carcinoma incidence and multiplicity in *K5-UCP3*/*K5-Akt* mice. Given the global metabolic changes caused by UCP3 overexpression, we speculate that these effects on tumour progression may be a result of, pleiotropic mechanisms affecting additional signalling molecules, however, we chose to focus on signalling pathways that are well known to play an important role in tumour promotion by TPA. We believe that the data make a strong case for Akt inhibition as a central mechanism in UCP3-induced resistance to tumour formation.

The role of UCPs in carcinogenesis is controversial: while some studies have reported that upregulation of UCP2, a close UCP3 homologue, confers tumours a survival advantage^41^,^42^, others have shown that UCP2 expression negatively impacts tumorigenesis^43^,^44^,^45^,^46^. The only two published cancer studies in UCP2 null mice have shown opposite effects of UCP2 knockout in the skin and colon^44^,^47^. We have previously shown that expression of UCP2 kills malignant cells but not a non-tumorigenic fibroblast cell line^48^. Furthermore, as shown in Supplementary Fig. 1c, animals that express an epidermal UCP1 transgene (*K5-UCP1*) are also resistant to two-stage chemical skin carcinogenesis, and mice expressing UCP1 specifically in skeletal muscle are protected from the development of lymphomas and other age related diseases^49^. Supporting our data, a recent report correlated UCP3 expression with Akt downregulation in a high throughput assay for FOXO1 nuclear localization^50^. Analysis of UCP3 expression in the Oncomine database revealed 17 studies in which UCP3 was among the top 10% of downregulated genes in cancer tissues compared with normal tissue controls (Supplementary Table 2). The present study establishes a molecular mechanism for UCP3-induced cancer prevention, and shows that enforced UCP3 expression blocks Akt activation in mouse and human keratinocytes.

The data herein support the argument that mitochondrial uncoupling may be a uniquely powerful metabolic intervention because it simultaneously targets a broad repertoire of interconnected metabolic and signalling changes essential for tumorigenesis. Moreover, tumour cells often exhibit profound metabolic flexibility, using a variety of nutrient sources for both energy production and biosynthetic reactions and as a result, many cancers are refractory to singly targeted metabolic therapies. Mitochondrial uncoupling likely restricts this flexibility by enforcing the constitutive oxidation of substrates, without concurrent ATP production. As our data indicate, uncoupled cells scavenge non-traditional nutrient sources, simultaneously limiting both the intermediates available for biomass production, along with important second messengers for growth signalling. Ultimately, the pleotropic effects of mitochondrial uncoupling provide a strategy to target both cancer cell metabolism and signalling, and may be a novel and effective therapeutic approach that circumvents many of the toxic manifestations of traditional chemotherapeutics.

## Methods

### Reagents

TPA and OA were purchased from LC Laboratories (Woburn, MA). Etomoxir ethyl ester (Eto) was purchased from US Biological (Salem, MA). Trypsin, Trypsin-EDTA, Keratinocyte Serum Free Media (KSFM), Hank’s Balanced Salt Solution (HBSS), dispase, gentamicin, fungizone, TMRM, MTG and Lipofectamine 2000 were purchased from Life Technologies (Grand Island, NY). Acetone, 7,12-dimethylbenz[*a*]anthracene (DMBA), and all other reagents (unless otherwise noted) were purchased from Sigma (St. Louis, MO).

### Animals

*K5-UCP3* FVB/N and *K5-Akt* FVB/N mice were previously generated using a bovine keratin 5 (K5) targeting construct as previously described^18^,^33^,^51^, maintained as hemizygous breeder colonies, and crossed *K5-UCP3* × *K5-Akt* to produce bitransgenic *K5-UCP3*/*K5-Akt* mice and littermate controls. FVB/N mice were purchased from Jackson Laboratories (Bar Harbor, ME). *Tg.AC* mice were purchased from Taconic (Hudson, NY). Unless otherwise indicated, all experiments were performed using sex matched adult, 6–8-week old mice. All animal husbandry and experiments were carried out in strict accordance to guidelines defined by the Association for Assessment and Accreditation of Laboratory Animal Care and approved by the institutional animal research committees at The University of Texas at Austin and UT-MD Anderson, Science Park Research Division.

### Primary cell culture

All cell lines were cultured under standard conditions of 5% CO_2_, 37 °C. Primary mouse keratinocytes were collected from the dorsal skin of adult mice and cultured according to standard methods^52^. Briefly, mice were killed by isofluorane inhalation, shaved on dorsal surface, and treated with depilatory agent. Dorsal skins were excised, and subcutaneous fat was removed and discarded. Skins were then floated on 0.25% trypsin for 1 h at 37 °C followed by an additional hour at room temperature. Epidermis was divided from the dermis, minced with scissors and basal keratinocytes were separated by centrifugation in a 22.5% percoll gradient. Following isolation, primary cells were cultured in collagen-coated plates with Eagle’s Minimal Essential Medium-2+1% FBS (0.05 mM Ca^2+^) (Invitrogen, Carlsbad, CA)^52^.

Primary neonatal human keratinocytes were isolated and cultured in KSFM for no more than five passages. To isolate neonatal human keratinocytes cells, human neonatal skin biopsies were floated overnight on dispase solution (HBSS, 0.75% sodium bicarbonate, 100 mM hepes, 10 mg ml^−1^ dispase, 5 μg ml^−1^ gentamicin, 10 ng ml^−1^ fungizone, pH. 7.4) at 4 °C. Epidermis was separated from underlying tissue and placed in Trypsin-EDTA (0.25% trypsin, 1 mM EDTA) for 5 min at 37 °C, then quenched with Dulbecco’s Modified Eagle’s Medium (Corning Incorporated, Corning, NY))+10% FBS+0.1% gentamicin and centrifuged at 3,000*g* for 10 min. Epidermal tissue was resuspended vigorously in KSFM before plating^53^.

### Flow cytometry and ATP measurement

Primary mouse keratinocytes were harvested from the dorsal skin of adult mice as described above. For ATP measurements, 10,000 cells per well were placed in a 96-well plate and ATP levels were measured in triplicate biological samples using the ApoSENSOR ADP/ATP Ratio Bioluminescent Assay Kit (BioVision Inc., Milpitas, CA). For flow cytometry experiments, primary keratinocytes were resuspended in HBSS and stained with Nile Red (Sigma) at a final concentration of 5 μg ml^−1^ for total cellular lipid content, or TMRM at 40 nM and MTG at 200 nM for membrane potential and mitochondrial mass measurements. All flow cytometric analysis was performed in triplicate biological samples using a BD Biosciences FACS Calibur.

### Topical treatments

For all experiments involving topical application of chemicals, mice were shaved on dorsal skin 48 h before treatment. All experiments were performed on mice between 6 and 8 weeks of age unless otherwise indicated (see timelines for tumour experiments). Two to four animals per group were used for each triplicate experiment. Males and females were divided evenly among groups. Mice were treated topically with 200 μl acetone (vehicle control), 12.5 μg ml^−1^ TPA (2.5-μg dose), 25 mM OA (5-nmol dose), or 5 mg ml^−1^ Eto (1-mg dose) and killed at the indicated time points. Dorsal skin biopsies were collected and fixed for histology or epidermal tissue was collected and used for immunoblotting as described below.

### BrdU labelling and histology

Twenty-four hours after acetone or single TPA treatment, mice were injected intraperitoneal. with 10 mg kg^−1^ BrdU in sterile saline, then killed 30 min after injection. For 4 × TPA treatment, mice received bi-weekly treatments for 2 weeks (total of four treatments), and were injected with BrdU and killed 48 h after the final TPA treatment. Dorsal skin biopsies were collected and fixed overnight in 10% neutral buffered formalin, moved to 70% ethanol and embedded in paraffin. Sections were stained as previously described^54^. Labelling index was calculated as the percentage of basal epidermal cells positive for BrdU. More than 100 cells from five randomly selected skin sections (total >500 cells) were counted from each of *n*=3 mice per genotype in each treatment group.

### Primary cell signalling experiments and transfection

For signaling experiments, mouse primary keratinocytes were grown in EMEM-2 with 1% FBS for 48–72 hours following isolation, then serum starved overnight (∼18 hours) before treatment. In NHK cells, transfections were performed using Lipofectamine 2000 reagent, and signaling experiments were performed 72 hours after transfection. NHK were moved to KSFM without epidermal growth factor (EGF) and bovine pituitary extract (BPE) supplements 18 hours prior to treatment. For experiments in both human and mouse cells, keratinocytes were treated with 40ng/mL EGF (Gemini Bioproducts, West Sacramento, CA) or 0.0001% BSA (vehicle control, Fisher Scientific, Pittsburgh, PA), incubated at 37°C, 5% CO2 until the indicated time point, then harvested and used for immunoblotting as described below.

### Immunoblotting

Epidermal tissue or whole cell lysates were prepared by lysis in RIPA buffer (50 mM Tris-HCl, 1% NP-40, 0.5% Na deoxycholate, 0.1% SDS, 150 mM NaCl and 2 mM EDTA) supplemented with protease and phosphatase inhibitor cocktails (Roche, Nutley, NJ). For membrane localization experiment, tissue fractionation was performed using differential centrifugation and density gradients^55^. Epidermal tissue was homogenized in 250 STMDPS Buffer (250 mM sucrose, 50 mM Tris-HCl (pH 7.4), 5 mM MgCl_2_, supplemented with protease and phosphatase inhibitor cocktails (Roche, Nutley, NJ)) using Dounce homogenizers with loose followed by tight fitting pestles. Homogenates were centrifuged at 800*g* for 15 min at 4 °C, after which supernatant was transferred to ultracentrifuge tubes and centrifuged at 100,000*g* for 1 h at 4 °C. Following ultracentrifugation, supernatant was saved as cytoplasmic fraction and pellets were re-suspended in ME Buffer (20 mM Tris-HCl (pH 7.8), 0.4 M NaCl, 15% glycerol, 1.5% Triton X-100 and supplemented with protease and phosphatase inhibitor cocktails) and saved as membrane fraction^55^.

Protein lysates were separated by SDS-polyacrylamide gel electrophoresis and transferred to nitrocellulose. Blots were probed with the following primary antibodies: α-phospho Akt S473 (Cell Signaling Technology (CST) 9271S, Rabbit, 1:1,000), α-phospho Akt T308 (CST 9275S, Rabbit, 1:1,000), α-Akt (CST 9272S, Rabbit, 1:1,000), α-phospho FOXO1 S256 (CST 9461, Rabbit, 1:1,000), α-phospho FOXO1 S319 (CST 2486, Rabbit, 1:1,000), α-phospho GSK-3β S9 (CST 5558, Rabbit, 1:1,000), α-GSK-3β (CST 12456, Rabbit, 1:1,000), α-phospho TSC2 S939 (CST 3615, Rabbit, 1:1,000), α-phospho mTOR S2448 (CST 5536, Rabbit, 1:1,000), α-phospho mTOR S2481 (CST 2974, Rabbit, 1:1,000), α-mTOR (CST 2983, Rabbit, 1:1,000), α-phospho 4EBP1 T36/47 (CST 2855, Rabbit, 1:1,000), α-4EBP1(CST 9644P, Rabbit, 1:1,000), α-phospho rs6 T240/244 (CST 5364. Rabbit, 1:1,000), α-rS6 (CST 2217, Rabbit, 1:1,000), α-phospho eIF4G S1108 (CST 2441, Rabbit, 1:1,000), α-phospho EGFR Y1086 (CST 2220S, Rabbit, 1:1,000), α-EGFR (CST 4267P, Rabbit, 1:1,000), α-phospho p38 MAPK T180/Y182 (CST 9211S, Rabbit, 1:500), α-p38 MAPK (CST 9212, Rabbit, 1:1,000), α-PTEN (CST 9188S, Rabbit, 1:1,000), α-PP2Ac (CST 2259, Rabbit, 1:1,000), α-PP2Aa (CST 2041P, Rabbit, 1:1,000), α-phospho ERK T202/Y204 (CST 4370, Mouse, 1:1,000), α-ERK (CST 9102, Rabbit, 1:1,000), α-phospho p90 RSK T359/S363 (CST 9344, Rabbit, 1:1,000), α-p90 RSK (CST 9347, Rabbit, 1:1,000), α-PKCα (CST 2056, Rabbit, 1:1,000), α-β-Actin (CST 4970S, Rabbit, 1:2,000), (CST, Danvers, MA), α-Cyclin D1 (Santa Cruz Biotechnology (SC)-753, Rabbit, 1:200), α-Cyclin A (SC-596, Rabbit, 1:200), α-p21 (SC-6246, Mouse, 1:200), α-p27 (SC-1641, Mouse, 1:200), α-α tubulin (SC-51500, Mouse, 1:200) α-α3 Integrin (AB1920, Rabbit, 1:500, Chemicon, Billerica, MA) and α-UCP3 (Rabbit, 1:2,000, custom made by Washington Biotechnology, Simpsonville, MD). Following primary antibody, blots were incubated with α-rabbit-horseradish peroxidase (HRP) (NA934V, Donkey, 1:3000, GE Healthcare, Piscataway, NJ) or α-mouse-HRP (NA931V, Sheep, 1:3,000, GE Healthcare, Piscataway, NJ), and developed using chemiluminescent substrate (Thermo Scientific, Rockford, IL). Results are representative of three separate experiments. Uncropped western blots are shown in Supplementary Fig. 8.

### PP2A activity assay

PP2A was immuno-precipitated using primary α-PP2Ac (CST 2259, Rabbit, 1:, Millipore, Billerica, MA), incubated with a target phospho-peptide and free phosphate release was measured via Malachite Green assay per the manufacturer’s instructions (Millipore, Billerica, MA).

### ROS measurement

ROS production by isolated mitochondria was measured using Amplex Red per the manufacturer’s instructions (Life Technologies, Grand Island, NY). Mitochondria from wild type and *K5-UCP3* epidermis were suspended in a buffer containing 5 mM MOPS (pH 7.4), 70 mM sucrose and 220 mM mannitol and mitochondrial protein concentration was determined using a BCA protein assay (Pierce Biotechnology, Rockford, IL). Mitochondrial protein (5 μg) per well were incubated in a reaction mixture containing 50 μM Amplex Red, 0.2 U ml^−1^ HRP, and 30 U ml^−1^ SOD at room temperature for 30 min, protected from light^56^. Superoxide dismutase was added to convert all superoxide into H_2_O_2_. Fluorescence was recorded using a microplate reader (VICTOR 3 V; Pelkin Elmer, Waltham, MA) with 531-nm excitation and 595-nm emission wavelengths.

2′,7′-dichlorodihydrofluorescein diacetate (Life Technologies, Grand Island, NY) and dihydroehidium (Life Technologies, Grand Island, NY) were used to detect cellular hydrogen peroxide and superoxide, respectively. Isolated primary keratinocytes were incubated with HBSS containing 5 μM 2′,7′-dichlorodihydrofluorescein diacetate or dihydroehidium. Fluorescence was analysed by flow cytometric analysis using a BD Biosciences FACS Calibur. Treatment with 10-mM succinate,10-μM antimycin A, or their combination was used to stimulate ROS production.

The redox state of Akt and PP2A was determined by analysis of cell extracts pre-incubated with AMS (4-acetamido-4′-maleimidylstilbene-2,2′-disulfonic acid). For AMS labelling experiments, cells were precipitated with 10% ice-cold trichloroacetic acid for 30 min at 4 °C and centrifuged at 12,000*g* for 30 min to collect precipitated protein. Protein pellets were re-suspended in 100% acetone and incubated at 4 °C for 30 min. Following centrifugation at 12,000*g* for 10 min, the acetone was removed and protein pellets were dissolved in 20 mM Tris/HCl, pH 8.0, containing 15-mM AMS and incubated at room temperature (25 °C) for 3  h (ref. 56). Akt and PP2A redox forms were separated by SDS-polyacrylamide gel electrophoresis in the presence of non-reducing loading buffer. Treatments with dithiothreitol or hydrogen peroxide were used to demonstrate reduced vs oxidized state of each protein.

### Metabolomic analysis

For metabolic profiling, mice (*n*=6 per genotype, age and sex matched) were fasted for 5 h before sample collection to control for metabolic variation due to feeding, then killed via cervical dislocation. Dorsal skin biopsies were taken and epidermal tissue was collected as described above. Samples were flash frozen in liquid N_2_ and stored at −80 °C until processed.

Unbiased metabolomic profiling analysis was performed by Metabolon (Durham, NC) according to their standard procedures^57^. Briefly, samples were prepared with the automated MicroLab STAR system from Hamilton Company using an aqueous methanol extraction process to remove proteins while allowing maximum recovery of small molecules. Aliquots of the resulting extract were analysed by ultrahigh performance liquid chromatography/mass spectrometry (UPLC/MS; positive mode), UPLC/MS (negative mode), or gas chromatography/MS. Raw data were extracted, peak identified, processed against quality control standards and normalized to total protein content by Metabolon. Compounds were identified by comparison with a library of >2,400 purified standards’ analytical characteristics on Metabolon’s LC and gas chromatography platforms. Compounds with a *P* value <0.05 in a Welch’s two-sample *t*-test were considered to be significantly different between genotypes.

### Fatty acid oxidation and oximetry

Endogenous fatty acid oxidation was determined in epidermal tissue in Kreb’s Ringer Buffer (Sigma), using a Clark-type electrode from Instech (Plymouth Meeting, PA). Saturated oxygen concentration was assumed to be 217 nMol/ml^−1^ for all readings. Respiratory rates were measured at linear regions on the curve under conditions of no treatment and 40-μM Eto. Fatty acid driven respiration was calculated as the basal respiration minus respiration after Eto treatment. To assess mitochondrial function in *K5-Akt* epidermis, respiratory rates were measured at linear regions on the curve under conditions of no treatment, 1 μg ml^−1^ oligomycin, and 250-μM 2,4-dinitrophenol to determine state 3, state 4, and maximal respiration, respectively.

### Gene ontology analysis

Gene expression analysis of *K5-UCP3* and wild-type dorsal skin was performed by Illumina microarray. RNA was isolated from untreated dorsal skin directly after isolation from 7-week-old male WT FVB and *K5-UCP3* mice (*n*=3) using TRIzol Reagent (Invitrogen, Carlsbad, CA) followed by a clean-up procedure using the QIAGEN RNeasy kit (QIAGEN, Inc.). Labelling was achieved by incorporation of biotin-16-UTP (Perkin Elmer Life and Analytical Sciences, Boston, MA) present at a ratio of 1:1 with unlabelled UTP. Labelled, amplified material (700 ng per array) was hybridized to the Illumina Mouse-6_V1 BeadChip according to the manufacturer’s instructions (Illumina, Inc., San Diego, CA). Arrays were scanned with an Illumina Bead array Reader confocal scanner and data processing and analysis was performed using the Illumina BeadStudio software. These data have been deposited in NCBI's Gene Expression Omnibus^58^ and are accessible through GEO Series accession number GSE71038.

Gene ontology analysis was performed using the functional annotation tool DAVID (http://niaid.abcc.ncifcrf.gov/home.jsp)^59^,^60^. Significantly up-regulated genes (calculated by Illumina DiffScore, a proprietary algorithm that builds an error model based on the bead s.d., *P*<0.01, DiffScore±22) in *K5-UCP3* dorsal skin were submitted to the DAVID website. Identified terms and gene names are shown in Supplementary Table 1.

### Tumour experiments

For *Tg.AC* tumour experiment, 16-week-old mice (*Tg.AC n*=20, *K5-UCP3*/*Tg.AC n*=20) were shaved dorsally and treated with bi-weekly applications of TPA (2.5 μg) for 2 weeks for a total of four treatments^61^. For *K5-UCP3*/*K5-Akt* rescue experiment, adult (6–8 week) WT *FVB/N* (*n*=28) and hemizygous *K5-UCP3* (*n*=27), *K5-Akt* (*n*=29) and *K5-UCP3*/*K5-Akt* (*n*=18) littermates were initiated topically on shaved dorsal skin with a single application of DMBA (100 μg) followed two weeks later with bi-weekly applications of TPA (2.5 μg) for 26 weeks^62^. In both experiments, sample size was chosen based on our previous empirical experience, which has shown that to detect differences of ∼30% in tumour burden with a power of 80 (at *P*=0.05) requires 25–30 mice/group (2-tailed unpaired *t*-test), while larger differences are detectable with smaller n. An even ratio of males and females between genotypes was ensured to control for sex-specific effects. Mice were scored weekly for tumour incidence (percentage of mice with skin tumours) and tumour multiplicity (number of skin tumours per mouse). Mice were killed if moribund, if any individual tumour reached a diameter of >1 cm, or at the termination of the experiment. Papillomas were distinguished histologically from carcinomas based on integrity of the basement membrane of the epidermis (invasion into the dermis and subcutis), overall cell morphology (well versus poorly differentiated) and the presence of other identifying characteristics (for example, keratin pearls, immune cell infiltration and angiogenesis).

### Statistics

Analysis of variance comparisons between genotypes were analysed by Student’s *t*-test or single factor analysis of variance followed by Dunnett’s *post hoc* test, with *P*<0.05 set *a priori* as statistically significant.

## Additional information

**Accession codes:** The microarray data have been deposited in NCBI's Gene Expression Omnibus and are accessible through GEO Series accession number GSE71038.

**How to cite this article:** Nowinski, S. M. *et al.* Mitochondrial uncoupling links lipid catabolism to Akt inhibition and resistance to tumorigenesis. *Nat. Commun.* 6:8137 doi: 10.1038/ncomms9137 (2015).

## Supplementary Material

Supplementary Information Supplementary Figures 1-8, Supplementary Tables 1-2 and Supplementary References
