# Supplementary material for: Mitochondrial uncoupling links lipid catabolism to Akt inhibition and resistance to tumorigenesis
Source: Nat Commun. 2015 Aug 27;6:8137. doi: 10.1038/ncomms9137 (PMC4552083; doi:10.1038/ncomms9137)
Supplement: Supplementary Information — Supplementary Figures 1-8, Supplementary Tables 1-2 and Supplementary References [file ncomms9137-s1.pdf]

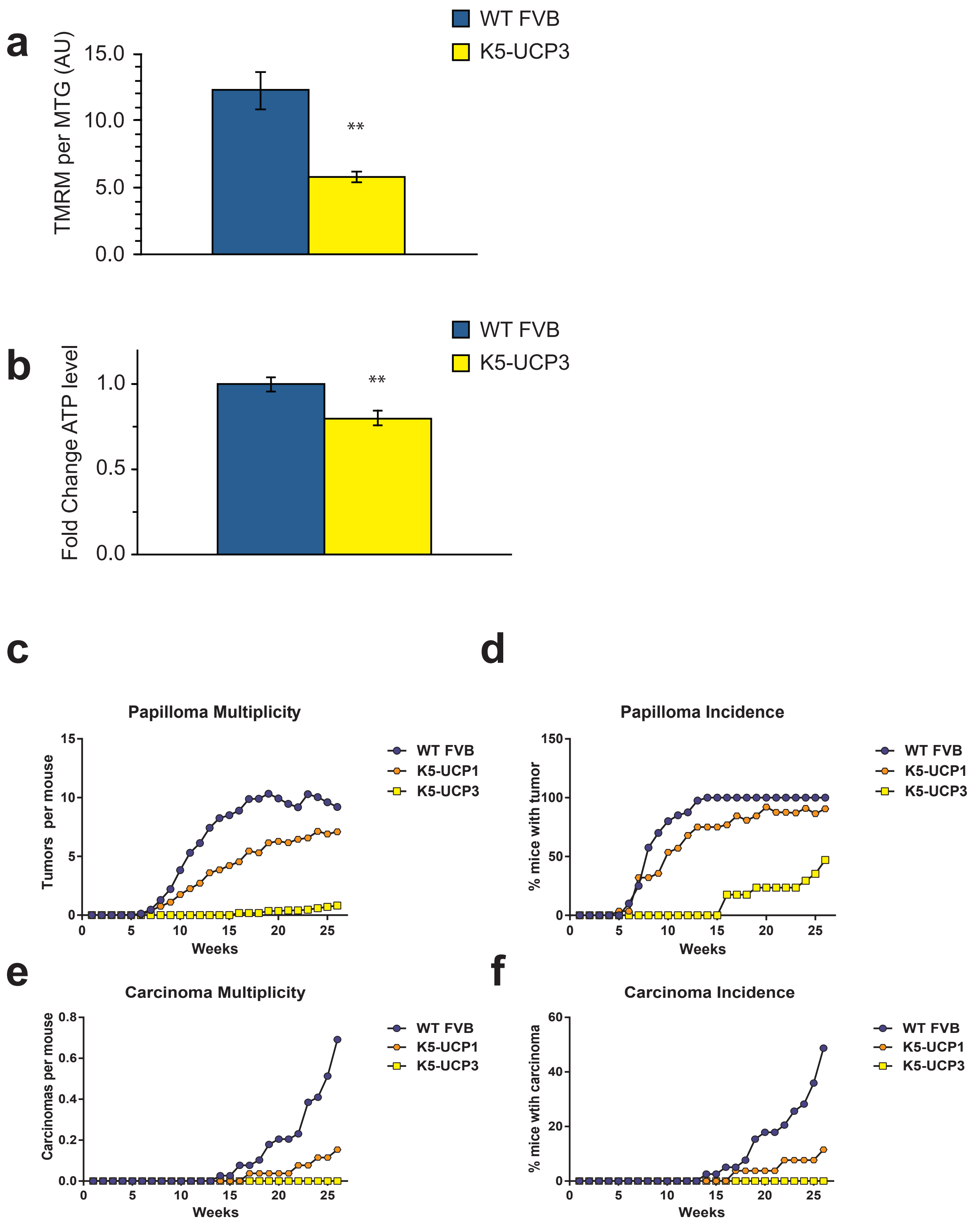

Supplementary Figure 1. Mitochondrial uncoupling is responsible for resistance to tumorigenesis. (**a**) FACS analysis of wild type and *K5-UCP3* isolated primary keratinocytes stained with TMRM (membrane potential indicator), normalized to MitoTracker Green staining (mitochondrial

mass indicator). Data represent mean fluorescence and error bars represent +/- SEM (n=3 biological replicates). (**b**) ATP levels in wild type and *K5-UCP3* primary keratinocytes as measured by bioluminescence assay. Error bars represent means +/- SEM (n=3 biological replicates). * indicates significantly different from wild type FVB, ($p<0.01$, student's t-test). (**c**) Tumor development in wild type FVB and *K5-UCP1* mice, indicating total papillomas/mouse, (**d**) % mice bearing papillomas, (**e**) total carcinomas/mouse, and (**f**) % mice bearing carcinomas. Previously published wild type and *K5-UCP3* data are shown for comparison[18].

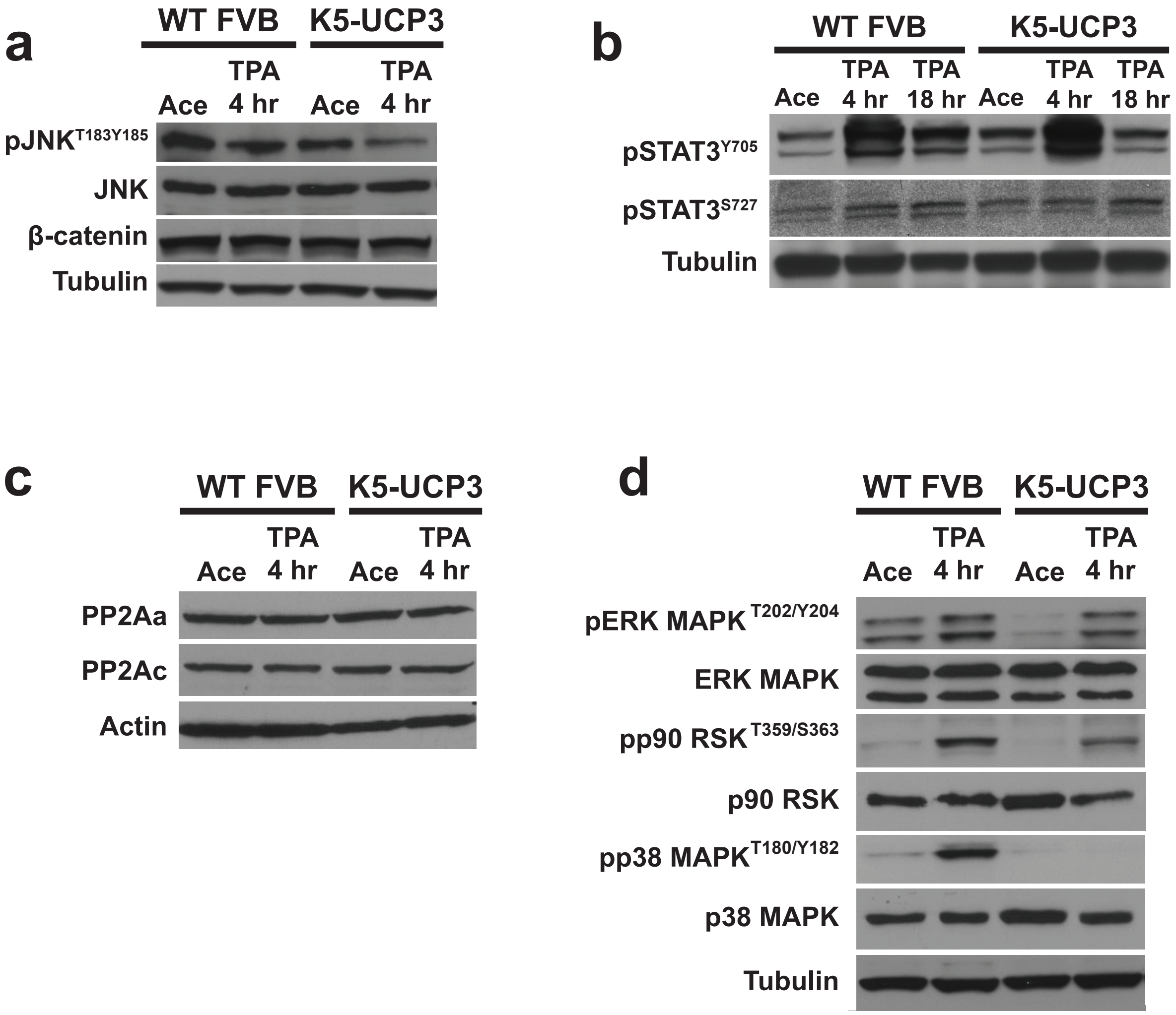

Supplementary Figure 2. PP2A is hyperactive in *K5-UCP3* epidermis. (**a**) Immunoblots for β-catenin, and pJNK. (**b**) Immunoblots for pSTAT3. (**c**) Immunoblot for expression of the A (scaffolding) and C (catalytic) subunits of PP2A. Immunoblotting for β-Actin was used to confirm equal loading. (**d**) Immunoblot for phosphorylation of additional PP2A targets, including pERK, p90 RSK, p38 MAPK. Immunoblotting for α-Tubulin was used to confirm equal loading.

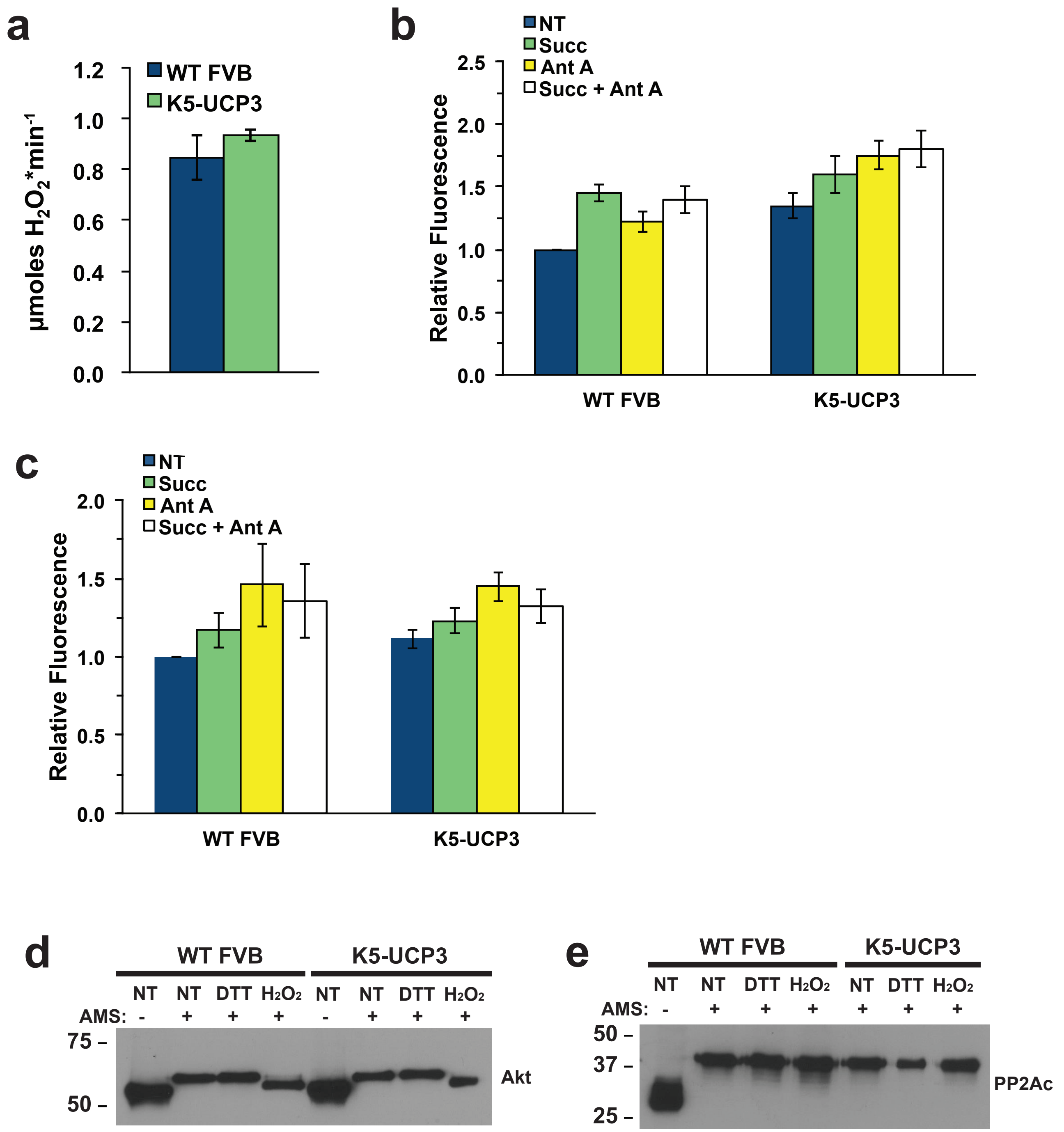

Supplementary Figure 3. Uncoupling has no significant effect on ROS in the *K5-UCP3* model.

(**a**) Amplex Red assay for rate of H_2O_2 production by mitochondria isolated from wild type FVB and *K5-UCP3* epidermis. (**b**) Dihydroxydichlorofluorescein and (**c**) dihydroethidium staining for cellular hydrogen peroxide and superoxide levels (respectively) in isolated primary mouse keratinocytes unstimulated, or treated with 10 mM succinate (Succ), 10 µM Antimycin A (Ant A), or their combination (Succ + Ant A). Data shown are fold change from mean wild type, unstimulated

fluorescence and error bars represent +/- SEM (n=3 biological replicates). (**d-e**) Immunoblots for thiol oxidation status of Akt (**d**) and PP2A (**e**). 4-acetamido-4′-maleimidylstilbene-2,2′-disulfonic acid (AMS) labels reduced cysteines, causing an increase in molecular weight and a resultant upward shift in mobility on the SDS-PAGE gel. Oxidation blocks AMS labeling, thereby blocking this upward shift. Treatment with dithiothreitol (DTT) and hydrogen peroxide (H_2O_2) were used as controls.

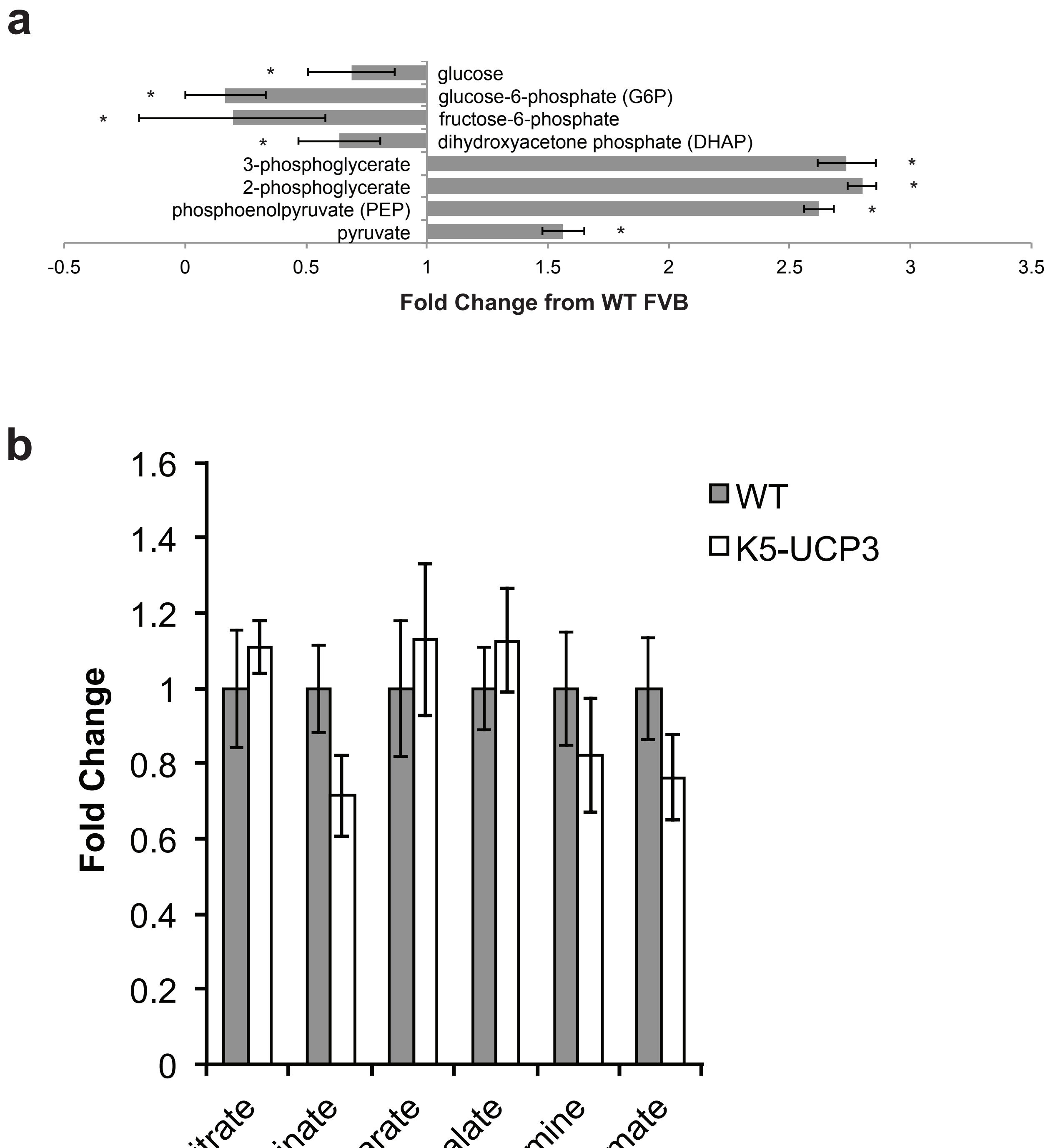

Supplementary Figure 4. Glycolysis, TCA cycle, and glutamine metabolites measured in unbiased metabolomic analysis of *K5-UCP3* epidermis. (**a-b**) Analysis of (**a**) glycolytic intermediates, (**b**) TCA cycle intermediate, and glutamine metabolite levels in *K5-UCP3* dorsal epidermis identified by gas chromatography mass spectrometry (GC-MS) or liquid chromatography mass spectrometry (LC-MS), and expressed as fold change compared to wild type FVB. * indicates

significantly different from wild type with a *p*-value <0.05 (Welch's t-test). Error bars represent means +/- SEM (n=6 animals per group).

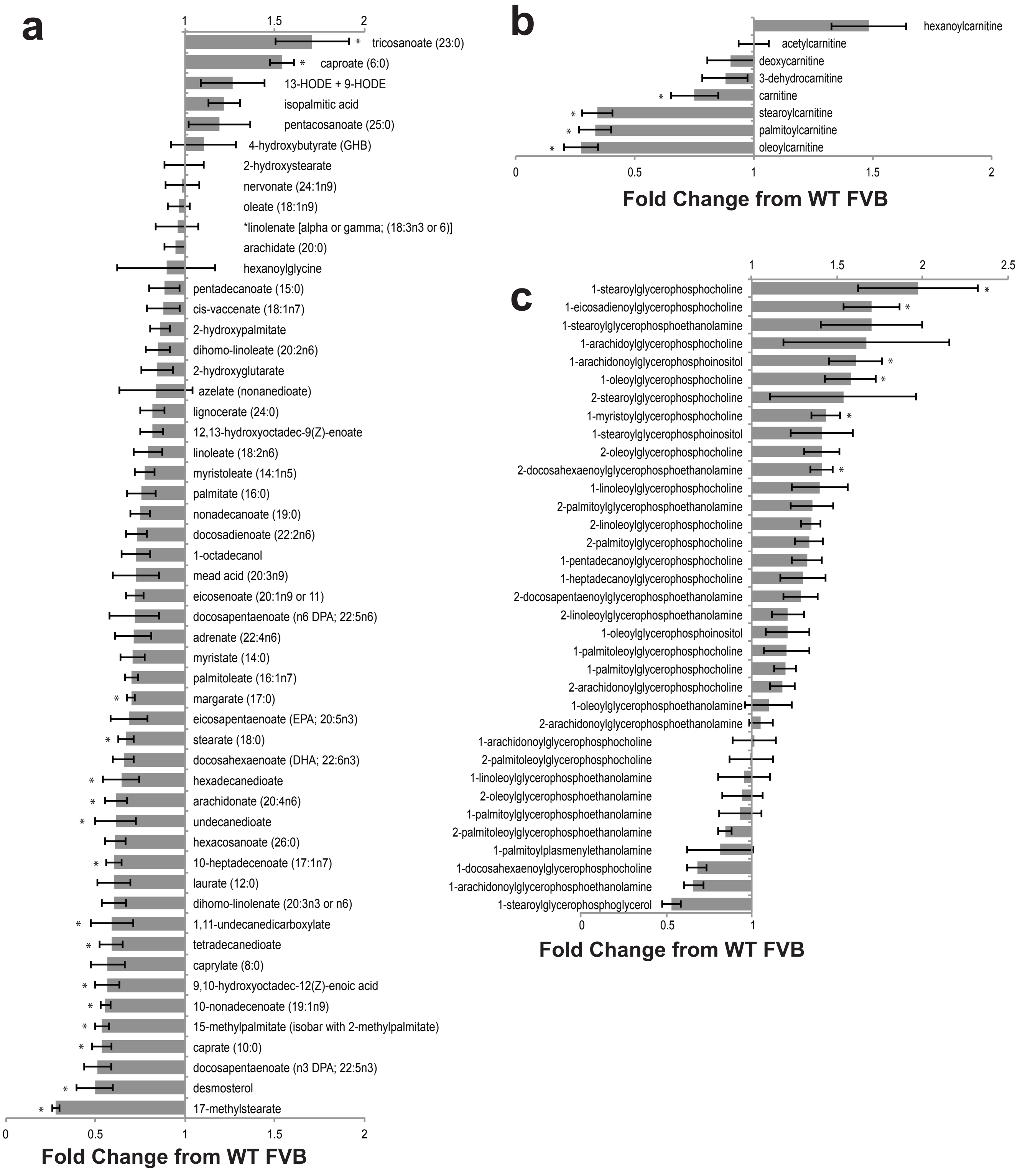

Supplementary Figure 5. All lipid species identified in unbiased metabolomic analysis of *K5-UCP3* epidermis. (**a-c**) Analysis of (**a**) fatty acid, (**b**) acylcarnitine, and (**c**) lysophospholipid metabolite levels in *K5-UCP3* dorsal epidermis identified by gas chromatography mass spectrometry

(GC-MS) or liquid chromatography mass spectrometry (LC-MS), and expressed as fold change compared to wild type FVB. * indicates significantly different from wild type with a *p*-value <0.05 (Welch's t-test). Error bars represent means +/- SEM (n=6 animals per group).

a

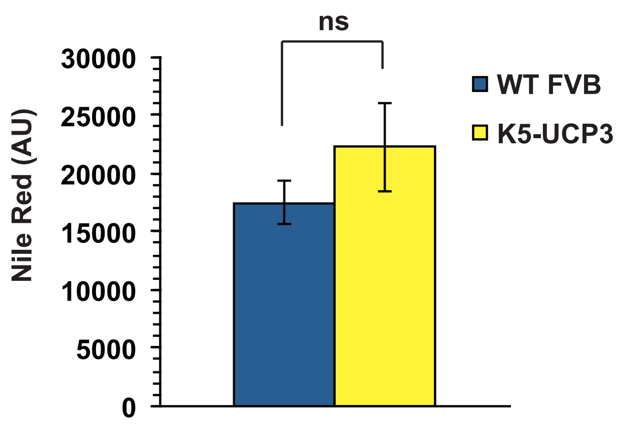

Supplementary Figure 6. UCP3 overexpression does not alter total cellular lipid levels. **(a)** FACS analysis of wild type and *K5-UCP3* isolated primary keratinocytes stained with Nile Red. Data shown are mean fluorescence and error bars represent +/- SEM (n=3 biological replicates). Ns denotes no statistically significant difference (student's t-test).

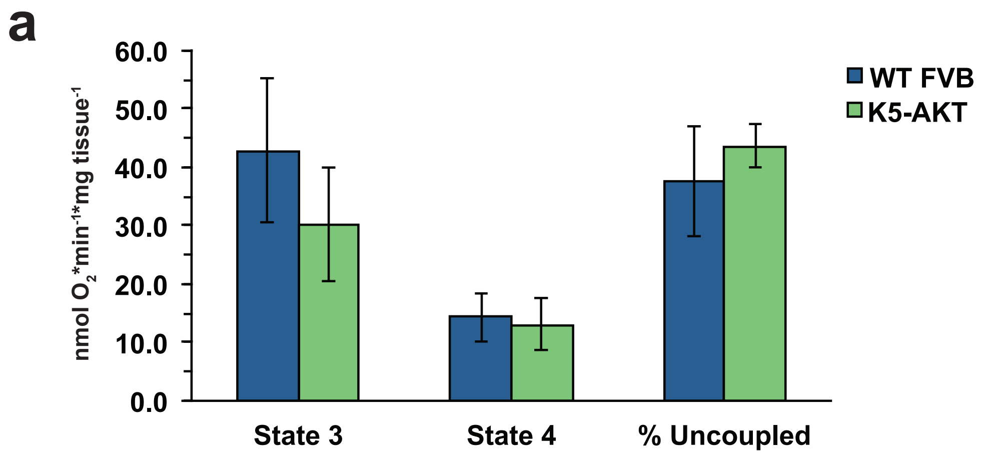

Supplementary Figure 7. Akt overexpression has no effect on epidermal respiration. **(a)** Oxygen consumption of isolated epidermis under no treatment, 1 µg/ml Oligomycin, and 250 µM 2,4-Dinitrophenol (DNP) to determine state 3, state 4, and maximal respiration, respectively. Error bars represent +/- SEM (n=5 biological replicates).

Full Westerns from Figure 2

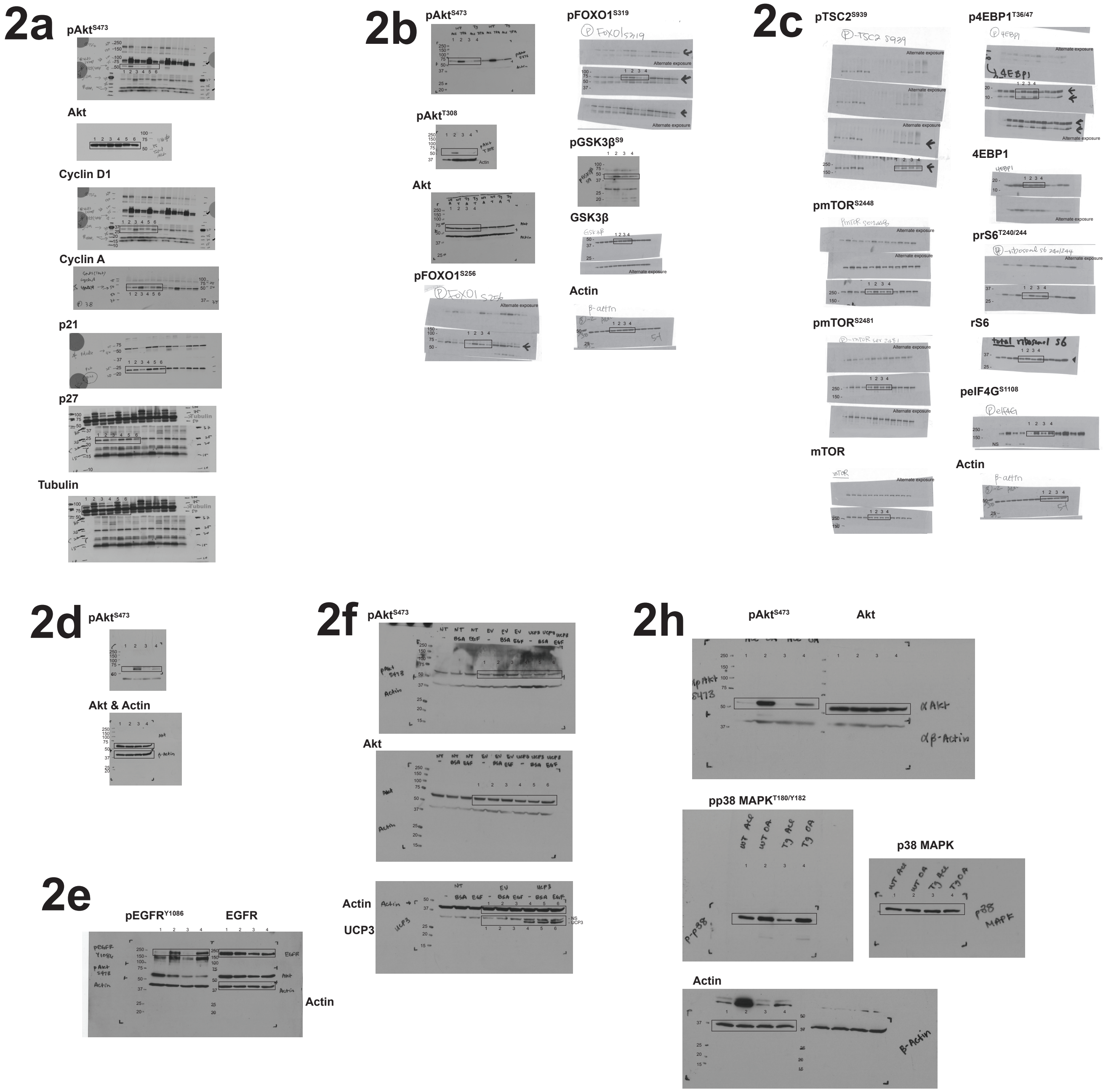

Full Westerns from Figure 4

4b

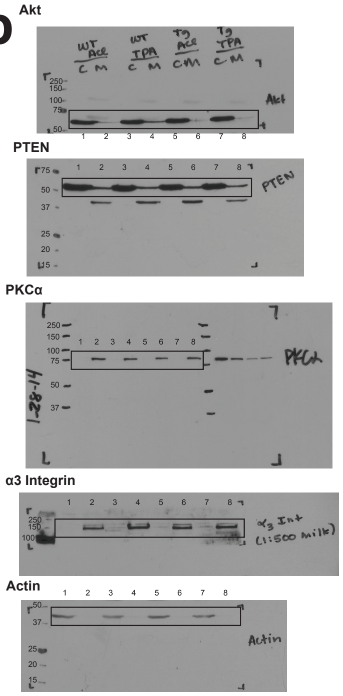

4d

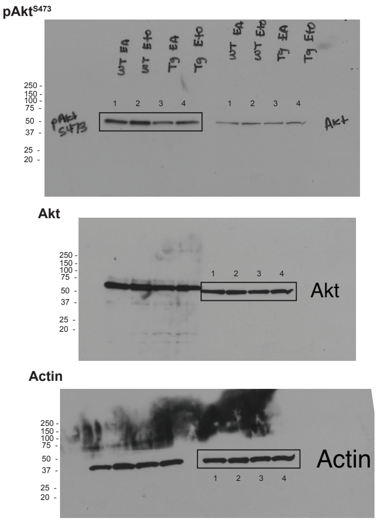

Full Westerns from Figure 5

5a

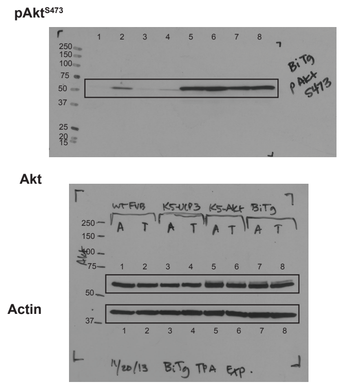

Full Westerns from Supp Figure S2

S2a

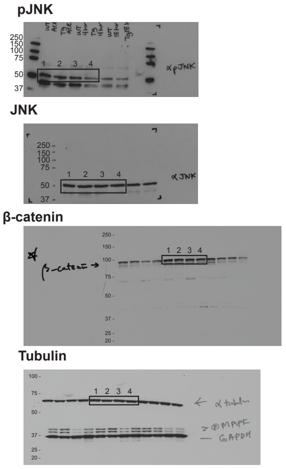

S2b

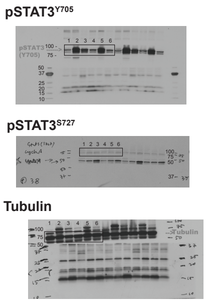

S2d

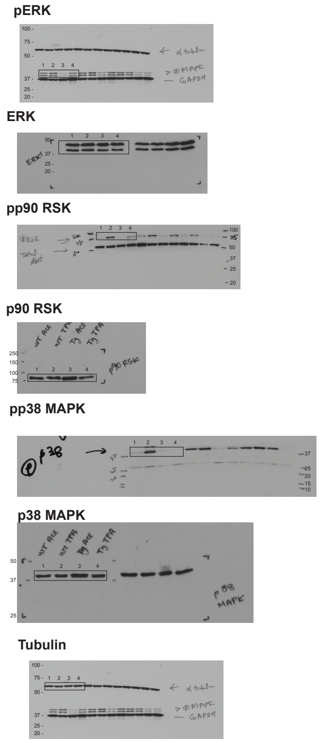

S2c

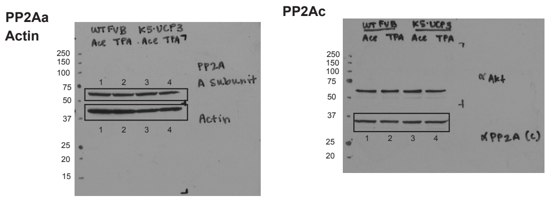

Full Westerns from Supp Figure S3

S3d

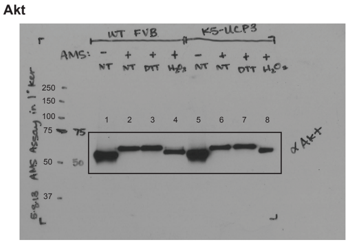

S3e

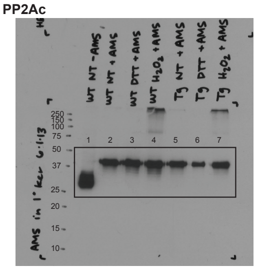

Supplementary Figure 8. Original images of full western blots. Black boxes denote the indicated lanes in figures.

Supplementary Table 1. Enrichment of lipid metabolic processes in *K5-UCP3* skin.

Category	Term	Official gene symbol	Gene name	Fold change	*p*-value
GOTERM_BP_FAT	Lipid biosynthetic process				1.3E-3
	20 counts	Dhcr24	24-dehydrocholesterol reductase	1.70	8.3E-3
		Dhcr7	7-dehydrocholesterol reductase	1.45	7.6E-3
		Stard5	StAR-related lipid transfer (START) domain containing 5	1.62	3.8E-4
		Ugcg	UDP-glucose ceramide glucosyltransferase	1.39	2.0E-3
		Alox12b	arachidonate 12-lipoxygenase, 12R type	1.85	6.4E-3
		Alox12e	arachidonate lipoxygenase, epidermal	1.94	2.4E-9
		Rnpep	arginyl aminopeptidase (aminopeptidase B)	1.34	2.4E-3
		Dgat1	diacylglycerol O-acyltransferase 1	1.57	3.0E-3
		Elovl3	elongation of very long chain fatty acids (FEN1/Elo2, SUR4/Elo3, yeast)-like 3	2.50	2.7E-6
		Elovl4	elongation of very long chain fatty acids (FEN1/Elo2, SUR4/Elo3, yeast)-like 4	1.33	1.9E-3
		Fads3	fatty acid desaturase 3	2.07	5.8E-3
		Insig2	insulin induced gene 2	1.70	7.9E-4
		Pigf	phosphatidylinositol glycan anchor biosynthesis, class F	1.70	1.5E-4
		Elovl6	predicted gene 11295; ELOVL family member 6, elongation of long chain fatty acids (yeast)	2.57	1.5E-5
		Rdh9	retinol dehydrogenase 9	1.28	1.9E-3
		Scarb1	scavenger receptor class B, member 1	1.45	2.6E-3
		Hmgcs1	similar to Hmgcs1 protein; 3-hydroxy-3-methylglutaryl-Coenzyme A synthase 1	1.36	3.2E-4
		Fdps	similar to farnesyl diphosphate synthetase; farnesyl diphosphate synthetase; predicted gene 5873; predicted gene 8163; predicted gene 3571	1.26	7.9E-3
		Scd2	stearoyl-Coenzyme A desaturase 2	1.50	9.2E-3
GOTERM_BP_FAT	Fatty acid metabolic process				1.7E-3
	15 counts	Aacs	acetoacetyl-CoA synthetase	1.53	5.5E-5
		Acsl4	acyl-CoA synthetase long-chain family member 4	2.23	2.1E-3
		Alox12b	arachidonate 12-lipoxygenase, 12R type	1.85	6.4E-3
		Alox12e	arachidonate lipoxygenase, epidermal	1.94	2.4E-9
		Rnpep	arginyl aminopeptidase (aminopeptidase B)	1.34	2.4E-3
		Elovl3	elongation of very long chain fatty acids (FEN1/Elo2, SUR4/Elo3, yeast)-like 3	2.50	2.7E-6
		Elovl4	elongation of very long chain fatty acids (FEN1/Elo2, SUR4/Elo3, yeast)-like 4	1.33	1.9E-3
		Faah	fatty acid amide hydrolase	1.57	3.4E-3
		Fads3	fatty acid desaturase 3	2.07	5.8E-3
		Lypla2	ysophospholipase 2	1.45	3.1E-7
		Mgst2	microsomal glutathione S-transferase 2	2.68	1.9E-5
		Elovl6	predicted gene 11295; ELOVL family member 6, elongation of long chain fatty acids (yeast)	2.57	1.5E-5

		Slc27a4	olute carrier family 27 (fatty acid transporter), member 4	1.62	2.3E-3
		Scd2	stearoyl-Coenzyme A desaturase 2	1.50	9.2E-3
		Tnfrsf1a	tumor necrosis factor receptor superfamily, member 1a	1.24	2.1E-3
GOTERM_BP_FAT	Lipid localization				1.8E-2
	10 counts	Abcg1	ATP-binding cassette, sub-family G (WHITE), member 1	1.79	1.9E-3
		Atp10d	ATPase, class V, type 10D	1.42	3.8E-3
		Gpihbp1	GPI-anchored HDL-binding protein 1	1.35	7.4E-3
		Stard4	StAR-related lipid transfer (START) domain containing 4	1.66	9.6E-8
		Stard5	StAR-related lipid transfer (START) domain containing 5	1.62	3.8E-4
		Lrp10	low-density lipoprotein receptor-related protein 10	1.35	1.5E-5
		Osbpl2	oxysterol binding protein-like 2	1.39	4.7E-4
		Osbpl3	oxysterol binding protein-like 3	2.24	1.1E-7
		Scarb1	scavenger receptor class B, member 1	1.45	2.6E-3
		Slc27a4	solute carrier family 27 (fatty acid transporter), member 4	1.62	2.3E-3
GOTERM_BP_FAT	Unsaturated fatty acid metabolic process				2.4E-2
	5 counts	Alox12b	arachidonate 12-lipoxygenase, 12R type	1.85	6.4E-3
		Alox12e	arachidonate lipoxygenase, epidermal	1.94	2.4E-9
		Rnpep	arginyl aminopeptidase (aminopeptidase B)	1.34	2.4E-3
		Mgst2	microsomal glutathione S-transferase 2	2.68	1.9E-5
		Tnfrsf1a	tumor necrosis factor receptor superfamily, member 1a	1.24	2.1E-3

Gene expression analysis of K5-UCP3 dorsal skin (7 week old male, n=3) was performed using the Illumina Mouse-6_V1 BeadChip array and analyzed using Illumina BeadStudio software. These data have been deposited in NCBI's Gene Expression Omnibus[1] and are accessible through GEO Series accession number GSE71038 (http://www.ncbi.nlm.nih.gov/geo/query/acc.cgi?acc=GSE71038). Gene ontology analysis was performed using the functional annotation tool DAVID (http://niaid.abcc.ncifcrf.gov/home.jsp)[2,3]. Significantly up-regulated genes (calculated by Illumina DiffScore, a proprietary algorithm that builds an error model based on the bead standard deviation, $p < 0.01$, DiffScore ± 22) in K5-UCP3 dorsal skin were submitted to the DAVID website. Identified terms and gene names are shown in Supplementary Table 1.

Supplementary Table 2. UCP3 is down-regulated in many forms of cancer.

Cancer type	Fold change	p-value	Reference
Pancreatic adenocarcinoma	-3.075	5.07E-07	Logsdon, C. D. *et al.* Molecular profiling of pancreatic adenocarcinoma and chronic pancreatitis identifies multiple genes differentially regulated in pancreatic cancer. *Cancer research* **63**, 2649-2657 (2003).
Tounge squamous cell carcinoma	-4.231	1.07E-05	Estilo, C. L. *et al.* Oral tongue cancer gene expression profiling: Identification of novel potential prognosticators by oligonucleotide microarray analysis. *BMC cancer* **9**, 11, doi:10.1186/1471-2407-9-11 (2009).
Glioblastoma	-1.441	5.67E-05	The Cancer Genome Atlas - Glioblastoma Multiforme Gene Expression Data (No associated paper, 2013/06/03)
Astrocytoma	-1.649	6.39E-04	Shai, R. et al. Gene expression profiling identifies molecular subtypes of gliomas. Oncogene 22, 4918-4923, doi:10.1038/sj.onc.1206753 (2003).
Oligodendroglioma	-1.679	1.54E-04	
Pleomorphic liposarcoma	-3.334	1.00E-03	Detwiller, K. Y. et al. Analysis of hypoxia-related gene expression in sarcomas and effect of hypoxia on RNA interference of vascular endothelial cell growth factor A. Cancer research 65, 5881-5889, doi:10.1158/0008-5472.can-04-4078 (2005).
Leiomyosarcoma	-3.673	2.91E-04	
Tounge carcinoma	-1.188	4.38E-05	Pyeon, D. et al. Fundamental differences in cell cycle deregulation in human papillomavirus-positive and human papillomavirus-negative head/neck and cervical cancers. Cancer research 67, 4605-4619, doi:10.1158/0008-5472.can-06-3619 (2007).
Oral cavity carcinoma	-1.34	3.53E-04	
Germinal Center B-Cell-Like Diffuse Large B-Cell Lymphoma	-1.428	2.69E-07	Compagno, M. et al. Mutations of multiple genes cause deregulation of NF-kappaB in diffuse large B-cell lymphoma. Nature 459, 717-721, doi:10.1038/nature07968 (2009).
Diffuse Large B-cell Lymphoma	-1.33	5.25E-11	
Activated B-Cell-Like Diffuse Large B-Cell Lymphoma	-1.3	3.04E-08	
Familial Parathyroid Hyperplasia	-1.288	3.00E-02	Morrison, C. et al. Molecular classification of parathyroid neoplasia by gene expression profiling. The American journal of pathology 165, 565-576, doi:10.1016/s0002-9440(10)63321-4 (2004).
Parathyroid Gland Adenoma	-1.354	7.00E-03	
Dedifferentiated Liposarcoma	-1.182	6.00E-03	The Cancer Genome Atlas - Sarcoma DNA Copy Number Data (No associated paper, 2013/05/23)
Angioimmunoblastic T-Cell Lymphoma	-1.245	3.75E-05	Piccaluga, P. P. et al. Gene expression analysis of peripheral T cell lymphoma, unspecified, reveals distinct profiles and new potential therapeutic targets. The Journal of clinical investigation 117, 823-834, doi:10.1172/jci26833 (2007).
Follicular Lymphoma	-1.224	4.00E-03	Brune, V. et al. Origin and pathogenesis of nodular lymphocyte-predominant Hodgkin lymphoma as revealed by global gene expression analysis. The Journal of experimental medicine 205, 2251-2268, doi:10.1084/jem.20080809 (2008).

This table describes the studies listed in the Oncomine database in which *UCP3* was identified as among the top 10% of down-regulated genes in the specified cancer type relative to normal tissue.

Supplementary References

1 Edgar, R., Domrachev, M. & Lash, A. E. Gene Expression Omnibus: NCBI gene expression and hybridization array data repository. *Nucleic acids research* **30**, 207-210, doi:10.1093/nar/30.1.207 (2002).

2 Huang da, W., Sherman, B. T. & Lempicki, R. A. Systematic and integrative analysis of large gene lists using DAVID bioinformatics resources. *Nature protocols* **4**, 44-57, doi:10.1038/nprot.2008.211 (2009).

3 Huang da, W., Sherman, B. T. & Lempicki, R. A. Bioinformatics enrichment tools: paths toward the comprehensive functional analysis of large gene lists. *Nucleic acids research* **37**, 1-13, doi:10.1093/nar/gkn923 (2009).
